# Benchmarking UMI-based single-cell RNA-seq preprocessing workflows

**DOI:** 10.1186/s13059-021-02552-3

**Published:** 2021-12-14

**Authors:** Yue You, Luyi Tian, Shian Su, Xueyi Dong, Jafar S. Jabbari, Peter F. Hickey, Matthew E. Ritchie

**Affiliations:** 1 Epigenetics and Development Division, The Walter and Eliza Hall Institute of Medical Research, 1G Royal Parade, Parkville, Australia; 2 Department of Medical Biology, The University of Melbourne, Parkville, Australia; 3 Australian Genome Research Facility, Victorian Comprehensive Cancer Centre, Melbourne, Australia; 4 Microbiological Diagnostic Unit Public Health Laboratory, Department of Microbiology and Immunology, The University of Melbourne at The Peter Doherty Institute for Infection and Immunity, Melbourne, Australia; 5 Single-Cell Open Research Endeavour (SCORE), The Walter and Eliza Hall Institute of Medical Research, 1G Royal Parade, Parkville, Australia; 6 School of Mathematics and Statistics, The University of Melbourne, Parkville, Australia

**Keywords:** scRNA-seq, Transcriptomics, Methods comparison, Sequencing analysis, Preprocessing

## Abstract

**Background:**

Single-cell RNA-sequencing (scRNA-seq) technologies and associated analysis methods have rapidly developed in recent years. This includes preprocessing methods, which assign sequencing reads to genes to create count matrices for downstream analysis. While several packaged preprocessing workflows have been developed to provide users with convenient tools for handling this process, how they compare to one another and how they influence downstream analysis have not been well studied.

**Results:**

Here, we systematically benchmark the performance of 10 end-to-end preprocessing workflows (*Cell Ranger*, *Optimus*, *salmon alevin*, *alevin-fry*, *kallisto bustools*, *dropSeqPipe*, *scPipe*, *zUMIs*, *celseq2*, and *scruff*) using datasets yielding different biological complexity levels generated by CEL-Seq2 and 10x Chromium platforms. We compare these workflows in terms of their quantification properties directly and their impact on normalization and clustering by evaluating the performance of different method combinations. While the scRNA-seq preprocessing workflows compared vary in their detection and quantification of genes across datasets, after downstream analysis with performant normalization and clustering methods, almost all combinations produce clustering results that agree well with the known cell type labels that provided the ground truth in our analysis.

**Conclusions:**

In summary, the choice of preprocessing method was found to be less important than other steps in the scRNA-seq analysis process. Our study comprehensively compares common scRNA-seq preprocessing workflows and summarizes their characteristics to guide workflow users.

**Supplementary Information:**

The online version contains supplementary material available at (10.1186/s13059-021-02552-3).

## Background

Over the past decade, single-cell RNA-sequencing (scRNA-seq) technologies and associated analysis methods have rapidly developed and been applied to a wide range of biological systems [1, 2]. The large number of analysis methods available presents a significant challenge for data analysts who are left to choose which of the many tools are best suited to their experiment and analysis goals. Fortunately, there are now many systematic benchmarking studies that explore this question in detail [3–6]. However, these evaluations almost exclusively focus on downstream analysis tasks, including normalization, clustering, trajectory analysis, cell type identification, and data integration. They ignore the crucial first step of preprocessing that summarizes the sequencing reads into a count matrix which is used as input to all downstream analyses.

The main difference between preprocessing scRNA-seq data compared with bulk RNA-seq lies in having to deal with various DNA barcodes which assist in the assignment of sequence reads to their cell or molecule of origin [7, 8]. Most methods are designed to work with unique molecular identifier (UMI) [9, 10] based data since these protocols are widely used in the field. UMIs are random oligonucleotide barcodes of a fixed length that are used to distinguish between the original molecules present in the cell and the PCR amplified copies generated during library construction. The process of UMI deduplication is a key part of transcript quantification in scRNA-seq data analysis that aims to provide molecule counts for the expressed genes in each cell and eliminate PCR-related quantification biases.

**Fig. 1:**
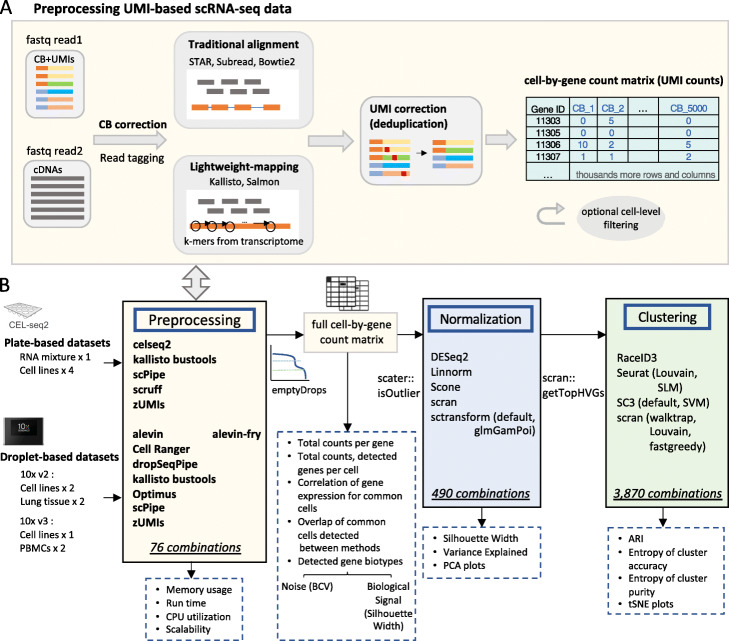
Overview of scRNA-seq preprocessing workflows and study design. (A) A typical preprocessing workflow begins with raw sequences in FASTQ files that are subject to cell barcode (CB) detection, alignment, UMI correction, count matrix generation, and quality control. (B) Summary of benchmarking study, showing the datasets analyzed, the selected preprocessing workflows and methods for normalization and clustering that were compared. Workflows and methods used in analysis are listed in boxes with solid borders, while evaluation metrics are shown in boxes with dashed borders. In total, 3870 combinations of datasets × preprocessing workflows × downstream analysis methods were generated in this study

Typical scRNA-seq preprocessing workflows involve demultiplexing, mapping, transcript quantification, and quality control. An overview of the main steps involved in preprocessing is summarized in Fig. 1 (A). Starting from raw FASTQ files, cell barcodes (CBs) and UMIs are first appended as tags to the header of each cDNA read. Next, cDNAs are aligned to either a reference genome or mapped using a lightweight method to the transcriptome depending on the particular pipeline. To overcome amplification bias, UMIs are collapsed to remove PCR-duplicated molecules from the gene counts in each cell. Base errors in CBs and UMIs are usually corrected at this step or before alignment. Next, reads are separated by CBs and assigned to genes or transcripts which allows the construction of a cell-by-gene count matrix (with cells in the columns and genes/transcripts in the rows). Next, cells of low quality and genes with low abundance are typically filtered out and the resulting count matrix is used in downstream analysis. Of note, CBs are known in advance for each well in plate-based protocols such as CEL-Seq [11] and randomly assigned to cells in droplet-based protocols, like 10x Chromium [12] and inDrops [13]. Because of this, different strategies are applied to construct CB “allow lists,” and additional steps to distinguish real cells from ambient RNA are suggested for droplet-based protocols [14].

Researchers can build their own preprocessing workflows by combining individual methods that address each of the aforementioned steps, or they may choose one of the many packaged workflows that have been proposed and published that aim to make this process more convenient. Examples of preprocessing workflows include *Cell Ranger* [12], *UMI-tools* [15], *scPipe* [16], and *zUMIs* [17]. More recently, alignment-free tools such as *kallisto* [18] and *salmon* [19] have been adapted to handle single-cell data to improve the computational efficiency of scRNA-seq data analysis. In addition, projects like the Human Cell Atlas [20] have developed their own preprocessing workflow (*Optimus* [21]) that uniformly processes the millions of human single-cell transcriptomes generated through this international collaboration. Other examples include the Single Cell Expression Atlas that has applied the *SCXA* pipeline (https://github.com/ebi-gene-expression-group/scxa-workflows) to process in excess of 4 million cells to build a large cross-species collection of single cell expression profiles [22].

Differences between published workflows arise when researchers balance efficiency with accuracy at each of the abovementioned steps. For example, to obtain CBs, most workflows use an allow list as a reference, whereas *salmon alevin* generates a putative list of highly abundant CBs that can be further filtered. To deduplicate UMIs, *kallisto bustools* [23] applied a naive collapsing strategy that they found to be more effective than more complicated approaches. Other pipelines such as *Cell Ranger* and *zUMIs* also take base quality and edit distance into consideration during UMI deduplication. Methods that place more importance on this step have also been developed after finding edit distances were inadequate for dealing with UMIs with high similarity [24, 25]. For example, *UMI-tools* introduced a network-based graph approach for this step, *alevin* [24] constructed parsimonious UMI graphs and *dropEst* [25] developed a Bayesian method to model UMI errors. To assign multi-mapped reads, several workflows, including *Cell Ranger*, *Optimus*, *dropSeqPipe,* and *kallisto bustools* discard them, while others treat ambiguous reads in different ways, assigning them to potential mapping positions probabilistically or via other strategies.

The small number of studies that have compared the performance of scRNA-seq preprocessing algorithms stops at the point before normalization after observing high correlations between count matrices obtained from different workflows or other custom combinations of methods for each step [17, 26]. Quantification differences were studied by comparing the performance of various popular alignment methods and annotation schemes [27] to help guide the choice of such tools. One study compared the performance of high-throughput scRNA-seq pipelines before and after normalization followed by clustering and differential expression analysis [28]. They pointed out a confounding factor akin to batch effects after integrating matrices processed by multiple different workflows applied to the same dataset. However, they did not utilize datasets with gold standard ground truth, and lightweight-mapping workflows such as *kallisto bustools* and *salmon alevin* were not included in the comparison. Two more recent studies [29, 30] compared the performance of the lightweight-mapping pipelines *bustools* and *alevin-fry* [31] in pseudoalignment mode. While their findings on the best-performing method in terms of running time and memory usage disagreed, they both concluded that similar downstream results were produced by these two approaches. In contrast, others found that *kallisto bustools* detects more cells with low gene content which are likely due to mapping artifacts [32], and pseudoalignment strategies can generate a number of false positive genes per cell [33]. Such spurious assignment was illustrated to be more severe when applying pseudoalignment and can be largely eliminated when structural constraints are applied, or when selective alignment is used [31, 33] as illustrated in *salmon*. Preprocessing tools have also been shown to influence the results of RNA velocity analyses [34] which highlights the potential for downstream effects driven by preprocessing algorithm choice. It is worth noting that previous comparative studies mostly focus on preprocessing for droplet-based protocols thus ignoring plate-based platforms, like CEL-Seq [11] and CEL-Seq2 [35] which are frequently used in some settings.

Here, we systematically benchmarked 10 end-to-end preprocessing workflows, including *scPipe*, *zUMIs*, *kallisto bustools*, *dropSeqPipe* [36], *Cell Ranger*, *Optimus*, *salmon alevin*, *alevin-fry*, *celseq2* [35] and *scruff* [37]. Among them, *celseq2* and *scruff* are specific to data generated by CEL-Seq and CEL-Seq2 protocols. *Cell Ranger* was developed for use with the 10x Chromium platform and is the standard workflow for 10x datasets. *DropSeqPipe* is only available for droplet-based protocols and is an unpublished online workflow with an instructional video on YouTube that teaches users how to run it. *Alevin* is a tool integrated within *salmon* that proposes new methods to handle UMIs and ambiguous reads. *Alevin-fry* is a successor to *alevin* that provides more options for mapping, cell detection, and quantification. *scPipe*, *zUMIs*, *salmon alevin*, and *kallisto bustools* can all handle raw data from both plate and droplet-based platforms, and the first three can also deal with Smart-Seq [38] (a full-length protocol that is not UMI-based) data.

We apply these methods to various scRNA-seq datasets with available ground truth containing varying biological complexity levels to benchmark their performance. Specifically, we describe the basic features of the count matrix produced by each preprocessing workflow and explore the impact on downstream analysis by evaluating the performance of combinations of preprocessing workflows together with various normalization and clustering methods using the *CellBench* platform [39].

## Results

### Benchmarking scRNA-seq preprocessing workflows

#### scRNA-seq preprocessing workflows evaluated

We investigated the performance of 10 end-to-end workflows applied to plate-based (CEL-Seq2) and droplet-based (10x Chromium v2 and v3 chemistry) data. To process CEL-Seq2 data, we applied *celseq2* and *scruff* (only applicable to plate-based protocols) along with *scPipe*, *zUMIs*, and *kallisto bustools* (applicable to both plate- and droplet-based protocols). For the 10x data we applied *dropSeqPipe*, *Cell Ranger*, *Optimus*, *salmon alevin*, *alevin-fry*, *scPipe*, *zUMIs* and *kallisto bustools*. Here *salmon alevin* was run using selective alignment to full genome decoys [40]. The *alevin-fry* method was run in pseudoalignment mode to the *splici* (spliced + intronic) reference [31]. Details of each workflow and the specific strategies applied during preprocessing are listed in Additional file 1: Table S1.

#### scRNA-seq datasets used for benchmarking

The *scmixology* datasets [5], which were designed for scRNA-seq benchmarking studies, include cells from distinct cell lines and provide ground truth in various forms (e.g., known clusters based on the mixing strategy applied or based on genetic variation between cell lines). These datasets involve experimental designs that use controlled mixtures of RNA (*RNA mixture*, 1 ×384-well plate of CEL-Seq2 data) to create “pseudo-cells,” or actual single cells from up to 5 human lung adenocarcinoma cell lines (4 ×384-well plates of CEL-Seq2 data and 2 ×10x Chromium v2 datasets). A new dataset using the same five cell lines profiled with 10x Chromium v3 chemistry was also generated (data available from GEO under accession number GSE154870). Other datasets included in our analysis were from the Tabula Muris project [41]. Cells from mouse lung tissue profiled by 10x Chromium (2 ×10x Chromium v2 datasets) were included to assess performance in a setting with more cellular diversity than the *scmixology* datasets. The final datasets which also included more cellular diversity were the 5k and 10k peripheral blood mononuclear cells (PBMCs) from healthy donor samples profiled using the 10x Chromium v3 chemistry. These data were downloaded from the 10x Genomics website. A summary of the datasets used, including the number of cells, expected number of clusters, and data structure is given in Additional file 2: Table S2.

Cell labels provided by the *scmixology* datasets were generated with intermediate BAM files created by *scPipe* based on single-nucleotide polymorphisms (SNPs) information for the cell line datasets and via labels available from the plate annotation for the mixture experiments. Cells from the Tabula Muris samples were manually annotated using canonical marker genes according to the approach described by the Tabula Muris Consortium (2018) [41]. Cells from the PBMC samples were manually annotated to specific cell types based on canonical immune cell markers (see the “Methods” section) using the same annotated cell types identified in another benchmarking study [33]. These annotated cell type labels were used as the ground truth in our study.

#### Benchmarking workflow

An overview of our benchmarking study design is presented in Fig. 1 (B). We generated a cell-by-gene count matrix using each of the preprocessing workflows listed in Additional file 1: Table S1. We performed cell-level quality control by firstly applying *emptyDrops* to distinguish empty droplets and cells (droplet-based protocols) and then using *scater* to identify and remove low-quality cells by setting a data-driven threshold on various quality control metrics (applied to both plate- and droplet-based protocols). Count matrices were normalized by six representative normalization methods that included *scran*, *Linnorm*, *scone*, *DESeq2* and *sctransform* (in either standard mode or using the method from the *glmGamPoi* [42] package). We then selected highly variable genes (HVGs) and applied up to eight commonly used clustering methods, including *RaceID3*, *Seurat* with the smart local moving (SLM) or Louvain algorithms, *SC3* in either default mode or with SVM, and *scran* with the walktrap, Louvain, or fastgreedy algorithms.

Overall, 3870 different combinations of datasets × preprocessing workflows × downstream analysis methods were obtained, with performance evaluated by several metrics at each step (see the “Methods” section and Fig. 1 (B)). *CellBench* was used to compare these combinations at the pipeline level, which allowed us to assess both the performance of a single method at a specific processing step and the interaction of multiple methods across several steps.

### Comparing computational performance of scRNA-seq preprocessing workflows

As single-cell technology develops, anywhere from hundreds to tens of thousands of cells are routinely profiled in an experiment. An important consideration when choosing between preprocessing workflows is their requirement of time and memory. Assessing how well different tools scale to datasets comprising of very large cell numbers when given more resources is another area of interest.

**Fig. 2:**
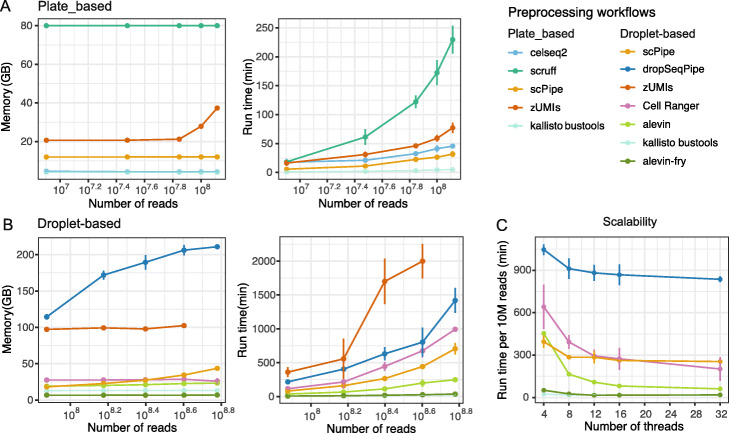
Comparing the computational performance of different scRNA-seq preprocessing workflows. Maximum memory usage and run time for each preprocessing workflow are shown for **A** plate-based protocols and **B** droplet-based protocols. Run time versus the number of threads is shown in **C**, where run time is scaled by 10 million reads

We compared workflows by specifying one node and eight threads on a High-Performance Computing (HPC) system. Maximum memory and time requirements were set for each submission. To control for competing workloads on the HPC, we ran each preprocessing method on datasets of different sizes three times per dataset and recorded run time, maximum memory usage, and CPU utilization (see the “Methods” section for details). A summary of the results obtained is shown in Fig. 2. For plate-based protocols, *scruff* required more memory and was slower when data volume increased (Fig. 2A), which is in contrast to the results of Wang et al. [37]. We speculate that this discrepancy is due to their use of smaller datasets containing fewer than 10 million reads and differences in the hardware and parallelization settings used for evaluation. *scPipe*, *zUMIs* and *celseq2* showed similar maximum memory consumption, running times and CPU utilization (Additional file 3: Figure S1A).

For droplet-based protocols, *zUMIs* was unable to provide results on FASTQ files sampled from the 10xv3_pbmc10k dataset with 600M reads within 2 days and had more variability in run time compared to other workflows on datasets that it did recover results for (Fig. 2B). Workflows based upon pseudoalignment tools required less memory resource and ran faster, which is concordant with previous studies [18, 19]. *Kalliso bustools* and *alevin-fry* were more than 50 times faster when dealing with 600M reads compared to the second slowest workflow *dropSeqPipe*. Maximum memory usage was at a similar level for the selected workflows except for *zUMIs* and *dropSeqPipe* which required considerably more memory. Among them, *dropSeqpipe* and *scPipe* used more memory as dataset size (in terms of the number of reads) increased. *Salmon alevin* and *alevin-fry* displayed the highest value of CPU utilization (Additional file 3: Figure S1B), indicating less time is spent waiting. Another study [28] also compared the computational performance of preprocessing workflows and demonstrated higher CPU utilization values and shorter running times for *Cell Ranger*, which is contrary to our results. This difference is likely due to our specification of a fixed number of cores and a limit on maximum memory usage for evaluation, whereas Gao et al. [28] did not.

In terms of scalability, workflows were run on the 10xv3_pbmc5k datasets, and the run time was scaled by 10M reads. As shown in Fig. 2C, *Cell Ranger* and *dropSeqPipe* displayed decreasing trends from 16 to 32 threads, suggesting they have better scalability, while the processing speed of *alevin-fry* saturated at 16 threads (Additional file3: Figure S1C) and *scPipe* and *kallisto bustools* saturated at 8 threads, which is consistent with another study [33].

### Comparing gene quantification across scRNA-seq preprocessing workflows

Besides computational efficiency, the characteristics and accuracy of the biological information recovered by different methods is another key consideration when selecting a preprocessing workflow. Using the cells retained after cell-level quality control (see the “Methods” section) and genes with overall expression above zero (i.e., a count of one or more in at least one cell), we characterized the workflows in terms of the number of genes detected per cell, total counts per cell, correlation of gene expression between common cells, and the concordance of retained cells identified by different workflows.

#### Gene quantification for CEL-Seq2 workflows

**Fig. 3:**
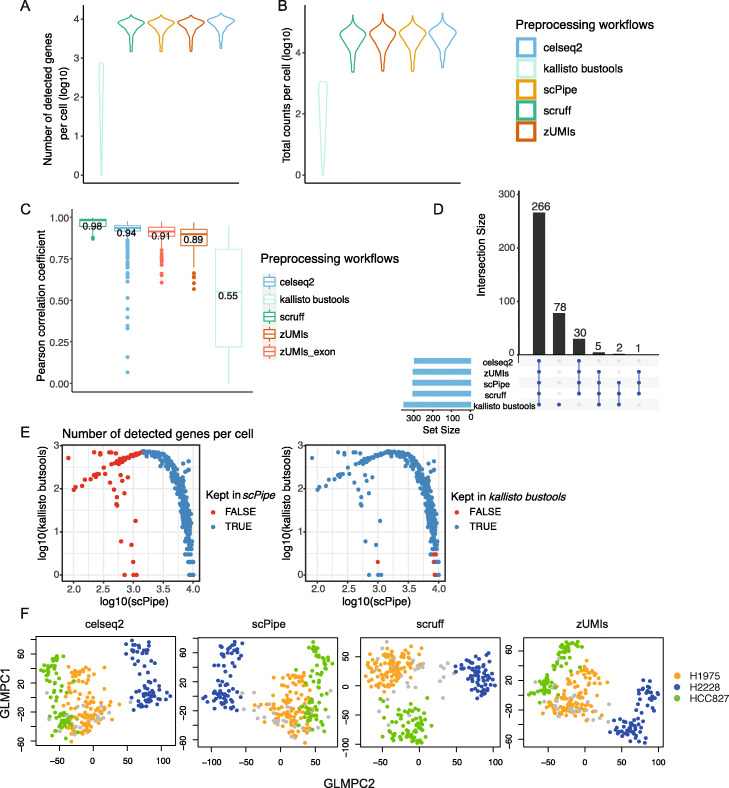
Comparing gene expression quantification of different scRNA-seq preprocessing workflows on the plate-based 3 cell line mixture (plate_3cell-line) dataset. **A** The number of detected genes per cell and **B** total counts per cell (both on a log10-scale). **C** The Pearson correlation coefficients between the gene counts of *scPipe* and other preprocessing workflows. Median values of the correlation coefficients are labelled. **D** After filtering, an *UpSet* plot displays the overlap of retained cells across workflows. **E** The number of detected genes per cell from *kallisto bustools* and *scPipe* are plotted in a pairwise manner. Colors represent whether a cell was kept after filtering with *scPipe* (left panel) and *kallisto bustools* (right panel). **F** GLMPCA plots for each preprocessing workflow, with colors representing the different cell lines included in this dataset. Cells that were not common between workflows are colored in grey

For the CEL-Seq2 benchmarking datasets, 5 preprocessing workflows were applied: *scruff*, *celseq2*, *scPipe*, *zUMIs,* and *kallisto bustools*. There was little variation in the different metrics assessed between CEL-Seq2 datasets, so representative results for the plate_3cell-line dataset are shown in Fig. 3.

In summary, the number of detected genes and total counts per cell showed high similarity across preprocessing workflows except for *kallisto bustools* (Fig. 3A, B), which recovered fewer genes and lower counts. Upon further investigation, this was found to be due to its strategy of only retaining unique CB-UMI pairs (further details on this issue can be found at https://github.com/BUStools/bustools/issues/44). For short UMIs (6 bp in the case of the CEL-Seq2 datasets), this limits the maximum detected features per cell to 4^6^=4,096 (3.61 on the log10-scale). In practice, the UMI counts are much lower, as whenever the same UMI is observed across more than one gene in a given cell, it is removed from the analysis altogether. This deficiency led us to exclude *kallisto bustools* from the majority of comparison plots for the CEL-Seq2 preprocessing results.

Slightly more detected genes per cell were observed with *celseq2* (Fig. 3A and Additional file 3: S2A), suggesting higher sensitivity of the aligner it uses (*Bowtie2*) on the datasets tested. *scruff* and *scPipe* were in complete agreement concerning the number of detected genes (Additional file 3: Figure S2A), which is as expected, as they share a very similar strategy across all preprocessing steps except for the quantification method used to count aligned reads. Higher total counts per cell were observed with *scPipe* compared with those of *scruff* (Additional file 3: Figure S2B). We speculate that the strategy applied in *scPipe* of UMI collapsing and read quantification may give rise to fewer collapsed UMIs and more assigned reads, and consequently, higher total counts per cell.

We next investigated the concordance of gene expression across workflows and compared the correlation of gene expression between *scPipe* and other methods using common cells and genes (Fig. 3C). Overall, we found relatively high average Pearson correlations (nearly all above 0.9), with correlations between *scPipe* and *scruff* the highest, whereas, with *celseq2* and *zUMIs*, the correlations were slightly lower (Fig. 3C). *Celseq2*’s use of a different aligner (*Bowtie2*) might account for the lower correlations, while for *zUMIs* the inclusion of intron reads in the gene counts is partially responsible, with the correlation increasing when run in exon-only mode. In terms of the overlap in cells detected by different methods, the majority (266) were common across all methods, with a further 38 found by at least 3 out of the 5 workflows tested (Fig. 3D). *kallisto bustools* detected 78 unique cells, which were deemed to be of low quality by other methods such as *scPipe* and filtered out due to low numbers of genes detected per cell (Fig. 3E).

Next, we applied *GLMPCA* [43], an alternative dimension reduction method for visualizing the raw counts from scRNA-seq data (Fig. 3F and Additional file 3: Figure S2C). Clear separation between cells from the different cell lines was observed for all preprocessing workflows, except for *kallisto bustools* (Additional file 3: Figure S2C). Samples from cell lines H1975 and HCC827 were observed to be more similar according to their bulk expression profiles in a previous study [44], which is broadly consistent with what we observe here at the single-cell level.

#### Gene quantification for 10x workflows

The same metrics were applied to the raw count matrix to compare the performance of 8 preprocessing workflows applicable to droplet-based 10x datasets (*scPipe*, *zUMIs*, *kallisto bustools*, *salmon alevin*, *alevin-fry*, *Cell Ranger*, *dropSeqPipe,* and *Optimus*) after applying *emptyDrops* or *Cell Ranger* v2 filtering (see the “Methods” section).

**Fig. 4:**
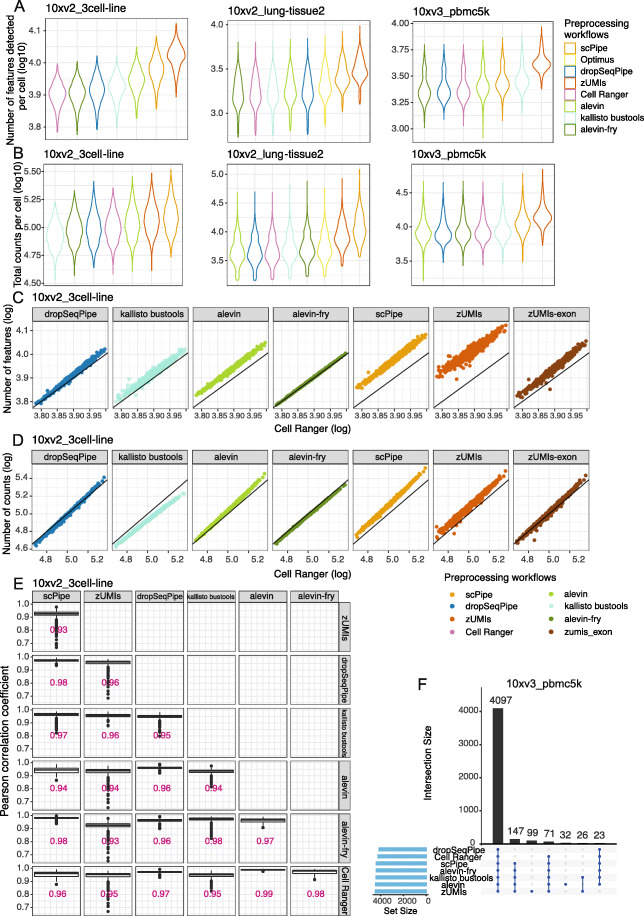
Comparing gene expression quantification of different scRNA-seq preprocessing workflows on droplet-based datasets. **A** The number of detected genes per cell and **B** total counts per cell (both on a log10-scale) on the 10xv2_3cell-line, 10xv2_lung-tissue2, and 10xv3_pbmc5k datasets. **C** The number of detected genes per cell and **D** total counts per cell for common cells for different preprocessing workflows against *Cell Ranger* on the 10xv2_3cell-line dataset. The identity line (*y*=*x*) is plotted in black in each panel. **E** The Pearson correlation coefficients between the gene counts of different pairs of preprocessing workflows for the 10xv2_3cell-line dataset. Median values of the correlation coefficients are labelled. **F** An *UpSet* plot displays the overlap of retained cells across workflows on the 10xv3_pbmc5k dataset

In terms of the number of detected genes and total counts per cell, *zUMIs* followed by *scPipe* systematically recovered more genes and higher counts across datasets (Fig. 4A, B and Additional filer~efMOESM3: Figure S3A-D), while results of other workflows are relatively concordant, except for *kallisto bustools* which detected more features on the PBMC datasets. To further explore these discrepancies, we compared the metrics obtained by *Cell Ranger* versus other workflows in a pairwise manner, with representative results on the 10xv2_3cell-line and 10xv3_pbmc5k datasets shown in Fig. 4C, D and Additional file 3: Figure S4A-B respectively. We summarized the differences in terms of whether there is a systematic overestimation or underestimation and the slope and variation of the linear relationships. Regarding the number of features detected, similar to the results of overall distribution of these metrics (Fig. 4A), *zUMIs*, followed by *scPipe*, *salmon alevin* on 10xv2_3cell-line, and *kallisto bustools* on 10xv3_pbmc5k recovered more features compared to *Cell Ranger*. Moreover, *zUMIs* and *kallisto bustools* displayed more variation from the fitted linear relationship. Including intron reads partially explained the extra detected features, as well as its variation (right-most panel Fig. 4C) for *zUMIs*, while for other workflows, applying a full reference annotation and different aligners should account for the extra genes detected compared to *Cell Ranger*, which uses a curated annotation. In terms of total counts per cell (Fig. 4D and Additional file 3: Figure S4B), *zUMIs*, followed by *scPipe* showed overestimation relative to *Cell Ranger* as well. Besides this, they generally recovered relatively more counts in cells with a higher abundance as quantified by *Cell Ranger* (slope of the fitted linear relationship above 1). This was also found with *salmon alevin*, *dropSeqPipe*, and *zUMIs_exon* on the 10xv2_3cell-line dataset. In contrast, *kallisto bustools* recovered higher counts in cells with lower content as quantified by *Cell Ranger* (slope below 1), which might be related to its naive UMI collapsing strategy. Interestingly, although *alevin-fry* uses a pseudoalignment approach, it provides the most concordant results with the least variation when compared against the *Cell Ranger* results.

Calculation of the Pearson correlation of expression for common cells across workflows showed high average pairwise concordance for all workflows, especially between *salmon alevin*, *alevin-fry*, *dropSeqPipe*, and *Cell Ranger* (Fig. 4E and Additional file 3: Figure S4C), with median correlation consistently above 0.95. *alevin-fry* also displayed a high correlation with *kallisto bustools*, which might be on account of them both applying pseudoalignment strategies. However, *zUMIs* showed relatively lower correlations with other workflows. Calculating correlations with intron counts excluded noticeably increased the correlation values (Additional file 3: Figure S4D), which suggests that the addition of intron counts is potentially adding noise to the results.

Next, *UpSet* plots were generated to assess the concordance of retained CBs after filtering across workflows (Fig. 4F and Additional file 3: Figure S4E). We found that most of the retained cells were common across workflows, and cells that were unique to a subset of the workflows made up less than 5% of the total number of cells.

### Comparing gene biotype detection across scRNA-seq preprocessing workflows

Genes of different biotypes can have systematically distinct length distributions and sequence similarity [45] and current RNA-seq tools have been shown to quantify genes possessing these characteristics differently [46, 47]. To explore differences in the detection and quantification of gene biotypes across scRNA-seq workflows, we investigated the signal and noise characteristics stratified by biotype.

#### Gene biotype detection for CEL-Seq2 workflows

**Fig. 5:**
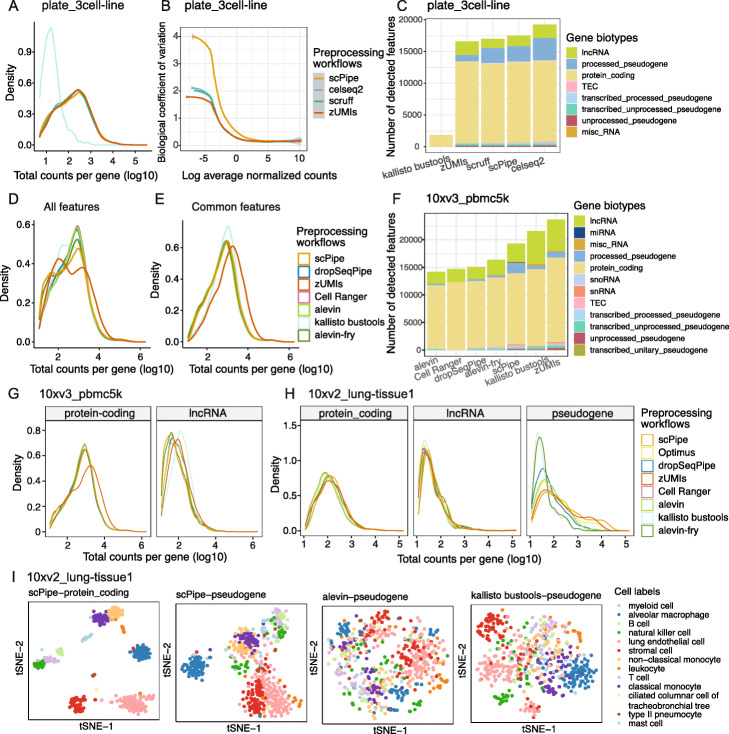
Comparing gene biotype detection of different scRNA-seq preprocessing workflows. **A** Density of total counts per gene (on a log10-scale), **B** biological coefficient of variation (BCV) for each feature versus gene abundance, and **C** the number of detected features per gene biotype for different workflows for the plate_3cell-line dataset. **D** The density of total counts per gene of all features, **E** common features (both on a log10-scale), **F** the number of detected features per gene biotype, and **G** the density of total counts (log10-scale) for distinct gene biotypes (protein coding genes and lncRNAs) for different workflows on the 10xv3_pbmc5k dataset. **H** Density plot for distinct gene biotypes (protein coding genes, lncRNAs and pseudogenes) for different workflows on the 10xv2_lung-tissue1 dataset. **I** tSNE plots generated with protein coding genes and pseudogenes using *scran* normalized counts for the 10xv2_lung-tissue1 dataset. Colors represent different cell type labels

For the representative plate_3cell-line dataset, density plots of the total counts per gene (Fig. 5A) were similar for all methods, except for *kallisto bustools*. To compare the biological noise for each workflow, the biological coefficient of variation (BCV) was calculated using the known cell labels from each dataset as the ground truth (see the “Methods” section). BCV measures the proportion of gene expression attributable to biological variability [48] and typically starts from higher values at low abundance and decreases monotonically as gene abundance increases. We observed such trends for most listed preprocessing workflows with selected biotypes using all cells and all features (Fig. 5B). The BCV trend for *scPipe* was systematically higher (especially for low intensity features) than the trends for other methods which are more consistent (results for *kallisto bustools* were excluded from this plot due to the limited numbers of features available to estimate BCV reliably). In terms of detected biotypes, the highest sensitivity and most lncRNAs were obtained with *celseq2*, which also detected features in the misc_RNA class (Fig. 5C), while *zUMIs* detected fewer pseudogenes compared to other methods. No major differences were observed in the count distributions of the various gene biotypes between different preprocessing methods (Additional file 3: Figure S5A).

#### Gene biotype detection for 10x workflows

Results for the droplet-based workflows (the 10xv3_pbmc5k dataset was chosen as a representative example in Fig. 5D and Additional file 3: Figure S5B, with other datasets featured in Additional file 3: Figure S5C-G) show bimodal gene count distributions for most datasets, with the exception of the lung tissue dataset (Additional file 3: Figure S5C) which has a unimodal count distribution. *Cell Ranger*, together with *salmon alevin* consistently detected fewer genes at the lower peak and more genes at the higher peak, whereas other methods provided relatively fewer features in the higher peak (Fig. 5D and Additional file 3: Figure S5D-G left panel). *zUMIs* and *kallisto bustools* tended to recover more lowly expressed genes across datasets, with *kallisto bustools* having a flatter density (on 10xv3_pbmc5k, 10xv3_pbmc10k, 10xv2_5cell-line) spread between the first and second peak observed using other methods.

If we restrict our analysis to features that are common across workflows, the lower peak is less prominent (Fig. 5E and Additional file 3: Figure S5D-G right panel), suggesting that the features quantified with low abundance tend to be discordant between workflows, which is in agreement with previous results [46]. After filtering the data in this way, the *zUMIs* peak is shifted from the left to the right and the density peak for *kallisto bustools* is much narrower.

We next studied the number of detected genes and proportion of counts assigned and found that they varied across different biotypes between workflows, with some workflows more likely to detect or assign counts to a particular class of genomic features than others (Fig. 5F and Additional file 3: Figure S6A). Most of the counts are assigned to protein coding genes across workflows (Additional file 3: Figure S6A). *Cell Ranger*’s use of a curated reference annotation restricts analysis to lncRNAs and protein coding genes, while *scPipe*, followed by *zUMIs* and *Optimus* assigned fewer counts to protein coding genes and a greater proportion of counts to pseudogenes. *Kallisto bustools* detected more lncRNAs (Fig. 5F) and generally assigned more reads to this class of features (Additional file 3: Figure S6A). Although short non-coding RNAs, including miRNAs, small nuclear RNAs (snRNA), and small nucleolar RNAs (snoRNA) were detected at very low levels (<0.2*%*, Additional file 3: Figure S6A), *scPipe*, *zUMIs,* and *Optimus* assigned a relatively higher percentage of UMI counts to these biotypes compared to other methods. The different biotypes influence the count distribution, with lncRNAs and pseudogenes contributing more to the lower abundance peak and protein coding genes to the higher abundance peak (Fig. 5G, H). Protein coding genes have similar densities, with a peak in the high abundance range across most methods except for *zUMIs*, where the peak is shifted to the right (Fig. 5G left panel and Additional file 3: Figure S6B-C left panel). This shift is also present for lncRNAs detected by *zUMIs* on the PBMC datasets (Fig. 5G right panel and Additional file 3: Figure S6B middle panel) and is presumably caused by the systematic inclusion of intronic and exonic reads when it assigns them to features. *kallisto bustools* shows a peak with higher abundance of lncRNAs as well on the PBMC and cell line datasets (Fig. 5G right panel and Additional file 3: Figure S6B-C middle panel), which might be the cause of its distinct distribution of total counts per gene observed when using all features (Fig. 5E and Additional file 3: Figure S5D-G, left panels).

The pseudogene total count per feature distributions were the most variable between workflows, with *kallisto bustools*, and *alevin-fry* having the sharpest left-skewed peak, suggesting more lowly expressed pseudogenes with fewer than 10 counts were quantified by these methods (Fig. 5H and Additional file 3: Figure S6B-C right panels). For other workflows, the peak is shifted to the right to varying degrees, indicating the existence of workflow-specific quantification biases for pseudogenes.

To further evaluate the quantification performance of selected workflows on pseudogenes, we examined long-read transcriptome sequencing data on the same cell line mixture samples [49] to obtain an independent measure of pseudogene abundance in the 10xv2_5cell-line and 10xv3_5cell-line datasets. Longer reads should map less ambiguously to pseudogenes compared to short-read data [50, 51], so the proportions obtained should be closer to the truth. Comparing the proportions of counts mapped to pseudogenes from the different preprocessing methods to those obtained in the long-read data, which themselves varied between the 10x v2 and v3 chemistry, we consistently observed fewer counts being assigned by *dropSeqPipe*, *salmon alevin* (selective alignment), and the pseudoalignment tools *kallisto bustools* and *alevin-fry*, which suggests that these workflows systematically underestimate pseudogene abundance (Additional file 3: Figure S6D-E). *zUMIs* recover pseudogene count proportions that are similar to the long-read estimates, while *scPipe* systematically assigns more reads to pseudogenes and is probably overestimating signal in this class of features.

### Comparing noise and biological signal across gene biotypes

Given the differences in detecting specific biotypes across the 10x preprocessing workflows compared, we next investigate the biological information captured by the 3 most abundant classes, which are protein coding genes, lncRNAs and pseudogenes (Additional file 3: Figure S6F). BCV and silhouette widths were calculated using the known cell type labels from each dataset as the ground truth (see the “Methods” section) to compare the biological noise and signal across biotypes for each workflow, respectively.

We observed expected BCV trends (i.e., BCV decreases as abundance increases) for all listed preprocessing workflows for the selected biotypes using common cells and all features (Additional file 3: Figure S7A-C left panel). BCV trends were similar for protein coding genes and lncRNAs for most of the datasets, while higher variance of lowly expressed genes were observed by *kallisto bustools*, *alevin-fry,* and *scPipe* on the lung tissue data; *zUMIs* on cell line data; and *scPipe*, *alevin-fry,* and *zUMIs* on the PBMC data. The trends were fairly distinct for the pseudogenes, especially for *kallisto bustools* and *alevin-fry* on the lung tissue data (Additional file 3: Figure S7A right-most side of left panel) and *salmon alevin* on the cell line data (Additional file 3: Figure S7B right-most side of left panel) where the BCV was systematically higher across the full range of abundance levels. The pseudogenes BCV trends for *kallisto bustools* and *alevin-fry* was markedly higher for the low abundance features on the PBMC datasets only (Additional file 3: Figure S7C right-most side of left panel). Restricting the analysis to common features and cells detected across all workflows saw similar trends (Additional file 3: Figure S7A-C right panel), although BCV values decreased overall compared to the results obtained from all features (Additional file 3: Figure S7A-C left panel), suggesting that quantification of discordant features is a major source of variation between workflows.

Next, we investigated the biological signal recovered by features from specific biotypes. Silhouette widths calculated on GLMPCs, which measures how similar a cell is to its “known” (pre-labeled) cell type compared to other cells were used and compared (see the “Methods” section). For the 10x cell line, lung tissue and PBMC datasets, protein coding genes showed similar and higher silhouette widths, indicating biological signal was universally retained by all workflows (Additional file 3: Figure S8A).

The separation between different cell types can be visualized using t-SNE plots created using protein coding genes (Fig. 5I left-most panel and Additional file 3: Figure S8B-C left-most panel). Most lncRNAs and pseudogenes had silhouette widths above 0 for the cell line datasets (Additional file 3: Figure S8A left column). However, on datasets with more cell type complexity, silhouette widths consistently above 0 were only observed for lncRNAs with *zUMIs* and for pseudogenes with *scPipe*, *Optimus,* and *zUMIs* on the lung tissue data (Additional file 3: Figure S8A middle column); lncRNAs with *alein-fry*, *dropSeqPipe*, *kallisto bustools,* and pseudogenes, with *scPipe* and *zUMIs* on the PBMC data (Additional file 3: Figure S8A right panel), suggesting that the separation between the known cell groups was less well defined by these feature types. *Kallisto* and *salmon* were recommended for detecting lncRNAs in a previous bulk RNA-seq benchmarking study [52] and although on the PBMC data we observe that *kallisto bustools*, *alevin*, *alevin-fry,* and *dropSeqPipe* recover more biological signal for lncRNAs than other methods, which is concordant with these findings, this was not seen in other datasets (Additional file 3: Figure S8A top row).

Additionally, looking at t-SNE plots based on pseudogenes for *salmon alevin*, *kallisto bustools,* and *alevin-fry* (Fig. 5I and Additional file 3: Figure S8B-D) shows a less clear separation between cell types compared to those provided by other workflows, especially on the cell line (Additional file 3: Figure S8C) and lung tissue datasets (Additional file 3: Figure S8D). This suggests that the quantification of pseudogenes by pseudoalignment or selective alignment using 3’ short-read sequencing data recovers less biological information. These results suggest that focusing analysis efforts on the signal from protein coding genes in datasets that profile complex tissues with greater cell type diversity may be an optimal strategy for all preprocessing methods.

### Comparing the effects of preprocessing workflows on downstream analysis

We next examined the degree to which the choice of processing workflow influences downstream analysis. Specifically, we look into how preprocessing impacts normalization, highly variable gene (HVG) selection, and clustering.

#### Comparing the performance of different combinations of preprocessing workflows and normalization methods

##### Normalization methods and evaluation metrics

Normalization has been shown to be an influential step in previous benchmarking studies [27, 53]. We applied six popular and well-proven normalization methods [5, 54], including *DESeq2* [55], *scone* [54], *scran* [56], *Linnorm* [57], and *sctransform* [58] (both the default method and updated strategy which uses *glmGamPoi* [59]), to explore how well different approaches remove any of the inherent biases introduced by preprocessing.

We evaluated the performance of each combination of dataset × preprocessing method × normalization algorithm using the silhouette widths of known cell groups, along with the amount of unwanted variation explained by library size and wanted variation explained by known cell groups on principal components (PCs). To summarize the results across the many different combinations of methods, linear models were fitted with silhouette widths as the response variable and the different methods as covariates. Higher silhouette widths and reduced unwanted variation are both indicators of better performance (see the “Methods” section). PCA plots were also generated for each combination of preprocessing workflow and normalization to assist in visualizing the expected data structure.

##### Normalization performance assessment on CEL-Seq2 datasets

**Fig. 6:**
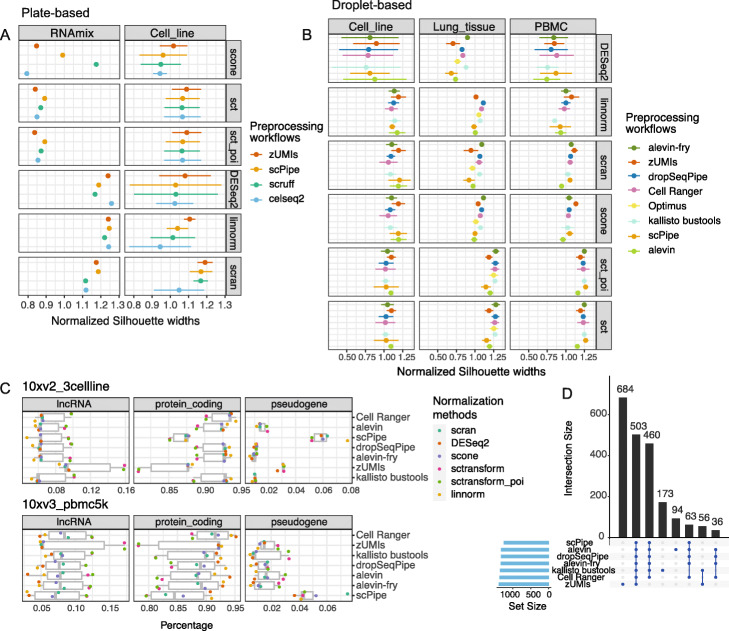
Comparing the performance of different scRNA-seq preprocessing workflows and normalization methods. **A** Dot plots (mean silhouette widths ± s.d) for plate-based datasets and **B** droplet-based datasets. Colors denote different preprocessing workflows. Silhouette widths are calculated based on known cell labels after applying different normalization methods and normalized against the silhouette widths obtained without any normalization. **C** The percentage of genes biotypes of lncRNAs, protein coding genes, and pseudogenes among HVGs on the 10xv2_3cell-line and 10xv3_pbmc5k datasets. **D** An *UpSet* plot displays the overlap in protein coding genes among the HVG list from different workflows obtained using *scran* normalized counts for the 10xv3_pbmc5k dataset

Considering silhouette widths on the CEL-Seq2 RNA mixture datasets, no preprocessing workflow systematically outperformed others when combined with different normalization methods (Fig. 6A left column). Example PCA plots are shown in Additional file 3: Figure S9A, with most of the combinations presented showing the expected trajectory paths, and combinations evaluated with higher silhouette widths displaying better separation between distinct RNA mixtures, e.g., for *scone*, *scruff* followed by *scPipe*, *zUMIs,* and *celseq2*. In terms of variation explained by library sizes, relatively less variation was explained by *scPipe* combined with any normalization methods compared to other preprocessing methods, which is preferred (Additional file 3: Figure S9B).

For the cell line datasets, *zUMIs* had slightly higher silhouette widths relative to other preprocessing methods while *celseq2* had consistently lower silhouette widths on average across different normalization methods (Fig. 6A right column and Additional file 3: Figure S9C). Regarding unwanted variation, performance was fairly similar across the different combinations (Additional file 3: Figure S9D).

##### Normalization performance assessment on 10x datasets

Similar silhouette widths after normalization were observed across workflows on the droplet-based datasets (Fig. 6B). On the cell line datasets, silhouette widths were higher for combinations of *scPipe*, *zUMIs,* and *salmon alevin* with *scran* or *scone* normalization. Overall, *zUMIs* followed by *salmon alevin* and *scPipe* provided slightly higher silhouette widths across all normalization methods, while *dropSeqPipe*, *kallisto bustools,* and *Cell Ranger* return relatively lower silhouette widths on the cell line data (Additional file 3: Figure S10A left panel). Inspection of PCA plots (Additional file 3: Figure S10B) from *scran*-normalized data showed that combinations with higher silhouette widths (*scPipe* and *zUMIs*) had better separation of H1975 and HCC827 cells compared to other workflows, although all methods showed good separation overall. In terms of unwanted variation, workflows performed fairly similarly, although *zUMIs* retained more library size variation post normalization than other methods (Additional file 3: Figure S10C).

On the lung tissue datasets, we observed that the best performing preprocessing methods on the cell line datasets (*zUMIs*, *scPipe,* and *salmon alevin*) tended to have slightly lower silhouette widths, while the other workflows performed fairly similarly, with slightly higher silhouette widths irrespective of the normalization method chosen (Fig. 6B middle column and Additional file 3: Figure S10A middle panel). In terms of unwanted variation, *scPipe* retained more library size variation than other methods and workflows that applied pseudoalignment (*alevin-fry* and *kallisto bustools*) in contrast retained less unwanted variation (Additional file 3: Figure S10D).

On the PBMC datasets, *zUMIs* performed better in combination with *scran*, *scone*, and *Linnorm*, while *scPipe* obtained higher silhouette widths when used together with *sctransform* normalization (Fig. 6B right column and Additional file 3: Figure S10A right panel). With respect to unwanted variation, *zUMIs* performed better as well with less library size variability, while *Cell Ranger* and *alevin-fry* performed relatively worse (Additional file 3: Figure S10E).

Although no preprocessing method consistently ranked the best across the datasets analyzed based on the silhouette widths (Fig. 6B), we found that the ranks of the preprocessing workflows across different normalization methods on datasets of a specific experimental design were relatively stable on average, suggesting that individual preprocessing workflows might incorporate quantification biases that cannot be eliminated by normalization.

##### Summary of normalization results

The performance evaluated by silhouette widths is summarized in Additional file 3: Figure S11. Results from all combinations of different preprocessing workflows and normalization methods show increased silhouette width compared to the results obtained without any normalization, suggesting that all normalization methods are highly effective on these data. In terms of preprocessing workflows, highly similar performance is displayed across methods, with normalization methods performing slightly better with *zUMIs* on droplet-based datasets, following very closely behind by *alevin-fry* and *dropSeqPipe*. On plate-based datasets the results are highly consistent between workflows, with less variation shown with *zUMIs*, followed closely behind by *scruff*, *scPipe* and *celseq2*.

#### Comparisons of HVGs selected on 10x datasets

After normalization, HVGs were selected for each combination of dataset × preprocessing method × normalization algorithm using *scran* (see the “Methods” section). Although the proportion of HVGs of different biotypes varies widely across datasets, protein coding genes account for the largest proportion of HVGs (Fig. 6C and Additional file 3: Figure S12A). Proportions also vary between preprocessing workflows, for instance *Cell Ranger* includes more protein coding genes than other workflows as would be expected since it excludes other biotypes, while *zUMIs* includes more lncRNAs and *scPipe* more pseudogenes. *zUMIs* also includes more pseudogenes among the HVGs on the cell line data compared to other methods and has similar proportions as *Optimus* on the lung tissue data.

From the perspective of how the choice of normalization method influences the HVG composition, we observe that more lncRNAs and fewer protein coding genes were included in the HVG lists obtained after applying *sctransform* irrespective of the preprocessing method, while *Linnorm* and *DESeq2* retained the most protein coding genes and fewest lnRNAs, which is especially pronounced for the PBMC datasets (Fig. 6C and Additional file 3: Figure S12A).

While most of the protein coding HVGs detected were common between workflows, distinct sets of genes of varying sizes were retained by individual preprocessing workflows in different datasets. For instance, on the 10xv3_pbmc5k data, *zUMIs*, *kallisto bustools,* and *salmon alevin* normalized by *scran* found 684, 173, and 94 unique genes respectively (Fig. 6D), while for *sctransform glmGamPoi* normalization, 555, 111, and 92 unique protein coding genes were recovered by these workflows (Additional file 3: Figure S12B). *zUMIs* also returned the largest list of unique lncRNAs after both *sctransform glmGamPoi* (Additional file 3: Figure S12B) and *scran* (Additional file 3: Figure S12C) normalization, followed by *kallisto bustools*. For pseudogenes, there were relatively few common highly variable features (8 or fewer, Additional file 3: Figure S12B-C), while *scPipe* selected the largest number of distinct pseudogenes (33 and 85 respectively) in its HVG list that were not retained by any of the other preprocessing workflows.

#### Comparing the performance of different combinations of preprocessing workflows and clustering methods

##### Clustering methods and evaluation metrics

There has been much research focused on the performance of clustering methods in terms of sensitivity of parameters, accuracy, robustness, etc. [53, 60, 61]. Here, we aim to investigate the impact of preprocessing on clustering instead of ranking clustering methods based on their performance. We selected representative clustering methods implemented in R and evaluated their performance when they reach the expected number of clusters based on the labels available. *RaceID* [62], *SC3* [63], *scran* and *Seurat* [64] were methods included. Both classic unsupervised methods and combined Support Vector Machine (SVM) methods in *SC3* were used. Graph-based clustering methods have been shown to perform fairly well previously, so *scran* with algorithms of fast-greedy, louvain, and walktrap and *Seurat* with louvain and SLM [65] were all included in the evaluation. The entropy of cluster accuracy (ECA), the entropy of purity (ECP) (see the “Methods” section), and adjusted Rand index (ARI) [66] were used for assessment of intra-cluster similarity, purity, and similarity of clustering partition with known clusters, respectively. ANOVA was then applied to assess the relative variation explained by the main analysis steps (preprocessing, normalization and clustering, see the “Methods” section). We also fitted a linear model to evaluate the extent to which specific methods or workflows at each analysis step, including preprocessing, normalization, and clustering, influenced the clustering result. ARI, ECA, or ECP were used as dependent variables in this analysis. Considering the intrinsic difference between experimental designs, ANOVA and linear models were fitted to the various combinations of methods separately for datasets with different designs.

##### Clustering performance assessment on CEL-Seq2 datasets

The clustering analysis for the RNA mixture datasets is more challenging compared to the other plate-based datasets due to the inbuilt trajectory paths which produce clusters that are very close to one another.

**Fig. 7:**
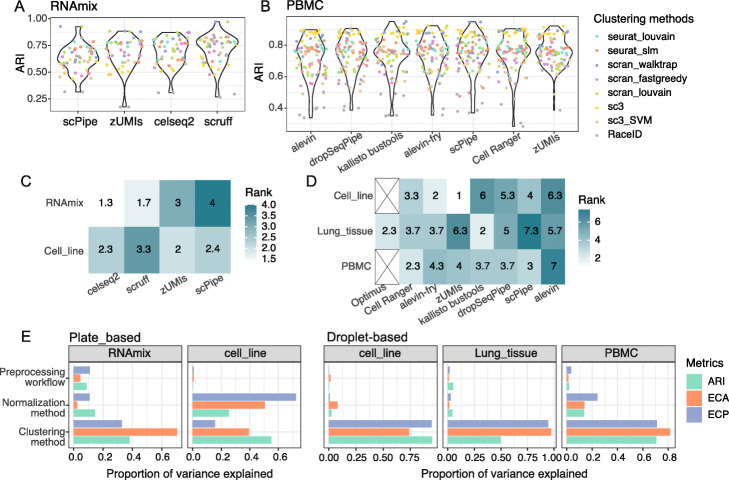
Comparing performance of different preprocessing, normalization, and clustering methods. **A** Violin plots of ARI for different preprocessing workflows on plate-based RNAmix dataset and **B** droplet-based PBMC datasets. Each point represents a method combination and is colored by the clustering method applied. **C** The preprocessing workflows’ influence on clustering results is summarized for plate-based data and **D** droplet-based data. Colors represent the rank of their average rank across evaluation metrics (ARI, ECA, ECP). Lighter color means better performance (i.e. higher rank). **E** Proportion of variance in ARI, ECA and ECP explained by the 3 major components of the analysis pipeline examined for plate-based (left) and droplet-based (right) datasets. Colors denote different performance metrics (ARI, ECA, and ECP) used as input to the ANOVA model (performance metric ∼preprocessing+normalization+clustering+experimental design)

In terms of ARI, combined with selected normalization and clustering methods, *scruff* produced more combinations with better performance, while *scPipe* provided relatively fewer and its average performance was relatively worse on the RNA mixture data (Fig. 7A and Additional file 3: Figure S13A). Combinations applying *scran* clustering algorithms delivered better results while those applying *SC3* have systematically lower ARI values. In terms of the top 3 combinations of entropy obtained for each preprocessing method, *scruff* tended to deliver better results compared to other workflows (Additional file 3: Figure S13B), and interestingly, no clustering method was observed to be consistently the best, while *Linnorm* normalization was the most common method among the top 3 combinations across workflows.

Overall, *scruff* and *celseq2* were ranked first and second across normalization and clustering method combinations according to the coefficients from the linear modeling, indicating better performance on average (Fig. 7C, top row). Inspection of t-SNE plots shows that combinations yielding better performance display clearer separation of cells from different mixture groups (*scruff*-*scran*-*scran_walktrap*), whereas method combinations that perform relatively worse have clusters made up of cells from multiple mixture groups (Additional file 3: Figure S13C-D).

For the cell line datasets, clustering is a much easier task since these comprise relatively fewer (3 or 5) well-separated clusters. On these data, nearly all method combinations obtained ARIs of 1 (Additional file 3: Figure S14A) and both ECA and ECP of 0 (Additional file 3: Figure S14B), except for *SC3* with SVM.

T-SNE plots of clustering results returned by different methods for the 3 cell line mixture dataset agree with the known cell types present (Additional file 3: Figure S14C). Overall, *zUMIs* ranked first, with *celseq2* and *scPipe* ranked equally second across all normalization and clustering method combinations using the linear model coefficients (Fig. 7C, bottom row). Considering the proportion of variance explained, we observed that for the RNA mixture datasets, clustering methods had a greater influence on performance (Fig. 7E left-hand panel), while for the cell line datasets, the choice of normalization method had a greater influence on the results (Fig. 7E right-hand panel). For both types of data, the preprocessing workflow explained the least variation in clustering performance (Fig. 7E).

##### Clustering performance assessment on 10x datasets

The 10x cell line mixture datasets also have a simple structure (3 or 5 expected clusters), and the performance of most method combinations, evaluated using ARI values, was 1 (Additional file 3: Figure S15A), with correct clustering of cells observed via t-SNE plots (Additional file 3: Figure S15B). In terms of ARI, *RaceID* had the lowest values, followed by *Seurat* (Additional file 3: Figure S15A). *Seurat* could not be coerced into producing the expected number of clusters, with an additional cluster of cells always retained, (Additional file 3: Figure S15B bottom row). We added up the number of combinations with both ECP and ECA at 0 and found that *scPipe* and *salmon alevin* delivered more optimal combinations, followed by *zUMIs*, indicating that these methods consistently delivered reliable results (Additional file 3: Figure S15C). On average, *alevin-fry* and *Cell Ranger* rank among the top 3 in terms of performance as estimated by the coefficients from the linear modeling (Fig. 7D top row).

For the lung tissue datasets, clustering results displayed similar performance, with comparable ARIs (Additional file 3: Figure S16A). In terms of top 5 combinations of entropy, *kallisto bustools* and *salmon alevin* delivered results with better performance compared to other workflows (Additional file 3: Figure S16B). Among these combinations, *SC3* was the most favored clustering method, followed by *scran* with the algorithm of fastgreedy across workflows. Overall, *kallisto bustools* and *Optimus* rank among the top 2 for performance as assessed by the coefficients from the linear model analysis (Fig. 7D middle row).

For the PBMC datasets, the clustering results were similar across preprocessing workflows when summarized by ARI (Fig. 7B and Additional file 3: Figure S17A). Clustering methods varied in performance, with *SC3* performing best, followed by *scran* with the algorithm of walktrap, while *RaceID* and *SC3* with SVM performed relatively worse (Additional file 3: Figure S17A-B). T-SNE plots agreed with ARI values, where combinations with higher values display clearer separation between known cell types, and clustering results that were concordant with the cell labels (Additional file 3: Figure S17C). In terms of the top 5 combinations of entropy, *scPipe* delivered results with better performance compared to other workflows (Additional file 3: Figure S17D), while results of *zUMIs* and *salmon alevin* were evaluated to have higher entropy. Among these combinations, instead of *SC3*, *scran* with the algorithm of louvain was the most favored clustering method across workflows. Overall, *Cell Ranger* and *scPipe* ranked among the top 2 for performance as assessed by the coefficients from the linear model analysis (Fig. 7D bottom row).

Consistent with observations from the plate-based datasets, the proportion of variation in performance explained in the cell line datasets, lung tissue datasets, and PBMC datasets was greatest for the clustering method while the variation in performance explained by preprocessing and normalization methods were both minimal (Fig. 7E right-hand panel).

## Discussion

### Summary of performance of preprocessing workflows

We compared the performance of 10 preprocessing workflows across CEL-Seq2 and 10x Chromium platforms on datasets with varying biological complexity and explored their quantification characteristics and impact on downstream analysis. In terms of preprocessing workflows designed for CEL-Seq2, the methods compared showed high concordance in quantification, with small discrepancies on detected features. Among them, *celseq2* is more sensitive and returned more non-protein-coding genes. Very similar results were returned by *scPipe* and *scruff* which share a lot of the same preprocessing choices, although more biological noise was observed in lowly expressed genes quantified by *scPipe* due to its UMI quantification approach. *scPipe* only collapses UMIs that differ by a hamming distance of 1 with more than a 2-fold difference in counts, which means the UMI counts obtained by *scPipe* tend to be relatively larger. In terms of downstream analysis, on the simpler (cell line) datasets, nearly all workflows produced clustering results that agreed well with the known cell type labels irrespective of the choice of normalization and clustering methods. On the more complex RNA mixture dataset, with clusters that are less well separated due to the gradient of mixture proportions used in this design, *scruff* and *celseq2* performed better (Fig. 7C). Our analysis revealed that *kallisto bustools* (v0.39.3) was unsuitable for use on CEL-Seq2 data due to its UMI handling strategy, which limits the dynamic range that can be observed in the presence of short UMIs (6bp in the case of these CEL-Seq2 datasets). This approach does not cause any noticeable feature detection issues in protocols that use longer UMIs such as 10x Chromium (10–12bp).

For the workflows compared on the 10x Chromium platform, several differences were observed with respect to the detection and quantification of genes. *Cell Ranger*, together with *salmon alevin* returned more features with higher abundance, which were mostly comprised of protein coding genes, whereas other workflows recovered more features at the second lower abundance feature peak. *kallisto bustools* delivered the most distinct count distribution with more features with relatively lower counts (around 100) or in the mid expression range (around 1000).

Also, we found that *alevin-fry*, *scPipe,* and *kallisto bustools* on the tissue datasets and *alevin-fry*, *scPipe,* and *zUMIs* on the PBMC datasets retained more biological noise, particularly among lowly expressed features compared to other workflows examined. Many of the lowly expressed features were not common between preprocessing workflows. Taken together, these differences indicate the uncertainty in detecting and quantifying lowly expressed features.

Previous studies have illustrated *Cell Ranger*’s bias for genes with low uniqueness [24] and the adverse effect of running it with a full annotation file [32]. *Cell Ranger*’s use of a targeted reference annotation that focuses on protein coding genes and lncRNAs and excludes biotypes that are more difficult to resolve with short-read sequencing, such as small RNAs and pseudogenes, would likely benefit other preprocessing workflows. The use of a modified feature set can not only alleviate multi-mapping issues but also avoid the workflow-specific gene quantification impacts seen in other gene biotypes that are not of principal interest (such as pseudogenes), although this approach would obviously not suit studies in which quantification of such features are of particular interest. We observed that biological signals present in the raw counts were nearly identical between *Cell Ranger* and the other preprocessing workflows compared. Additionally, after downstream analysis, we observed *Cell Ranger*’s performance to be consistently high and relatively stable, which supports the results of another recent study [32]. Some preprocessing workflows were found to be relatively more or less likely to assign counts to a particular class of genomic features than others. For example, *scPipe* assigned systematically more reads to pseudogenes, whereas *dropSeqPipe*, *salmon alevin*, *alevin-fry,* and *kallisto bustools* assigned many fewer reads to this feature class. In addition, for the workflows that use pseudoalignment or selective alignment, *kallisto bustools* provided more pseudogenes yielding a limited number of counts; *alevin-fry* and *salmon alevin* recovered more biological noise in pseudogenes.

After normalization, evaluating the performance of preprocessing workflows based on known biological information uncovered similar performance in all but a few method combinations, indicating high concordance between preprocessing workflows.

When selecting highly variable genes, interestingly, we found that features of different biotypes were present in varying proportions across workflows, with the choice of normalization method also influencing the proportions of different gene biotypes that made it into the HVG list.

Regarding clustering results, similar performance was observed across different workflows and methods on selected datasets. Clustering results that were highly consistent with the provided cell labels were observed on the cell line datasets, and on the lung tissue and PBMC datasets, where cellular complexity was higher, we still observed good performance across all workflows.

Overall, *Cell Ranger*, *alevin-fry* performed slightly better on droplet-based datasets on average. Although slightly lower correlations were observed between *zUMIs* and other workflows, and it uniquely considers intron reads by default, we did not observe improved clustering results by applying it.

Intron reads are indicated to be informative as they likely originate from nascent mRNA [67] and were shown to assist in extracting more information when included in quantification [17]. However, the scRNA-seq data we analyzed both rely on poly(A) selection, which may limit the amount of intron signal that can be extracted. A summary of the advantages and limitations of the preprocessing workflows evaluated is presented in Additional file 4: Table S3.

### Limitations

Our benchmarking study is subject to several limitations. First, although there are other protocols such as InDrops, Drop-seq, and Sort-seq, and different protocols are known to influence downstream analysis [26, 68], our results are restricted to datasets from two protocols, CEL-Seq2 and 10x Chromium. Second, we did not expand our analysis to include other important aspects of the single-cell data processing, such as dimensionality reduction and feature selection [53]. Extending our benchmarking to cover additional protocols, new preprocessing methods, and other data analysis tasks is left as future work.

Furthermore, the sample types studied are limited to cells from cancer cell lines, primary lung tissue, and PBMCs. Further work could explore the performance of preprocessing workflows on datasets that include a more diverse range of cell types, e.g., cells that make up the tumor microenvironment, although we would anticipate broadly similar results.

Another aspect not investigated in our study is which specific step within different preprocessing workflows has the most influence on performance. Although we observed quantification differences between workflows, we did not delve further into the individual steps within a workflow, which include alignment, deduplication, etc. Previous studies have already provided relatively comprehensive comparisons of alignment and quantification methods [27, 40]. Hence, the results from our benchmarking study are targeted more to workflow users rather than workflow developers.

## Conclusions

Our assessment investigated the quantification performance of preprocessing workflows and their impact on downstream analysis. We found that scRNA-seq preprocessing workflows varied in their detection and quantification of lowly expressed genes across datasets. However, after subsequent downstream analysis by well-performing normalization and clustering methods, even if the proportion of different gene biotypes detected differed and workflow-specific genes were identified within the various sets of highly variable genes, nearly all combinations delivered good performance with relatively minor differences in the final cell clustering results. Our detailed analysis of 3870 datasets × method combinations, made possible by the *CellBench* evaluation framework, finds that the choice of preprocessing workflow has relatively less impact on the results of a single-cell analysis than subsequent downstream analysis steps such as normalization and clustering.

## Methods

### Preprocessing workflows compared

We evaluated 10 publicly available preprocessing workflows in total (Additional file 1: Table S1). Workflows that started from raw FASTQ files and provide a cell-by-gene count matrix as output was chosen. All workflows were installed and run locally, except for *Optimus*, which was run on Terra (https://app.terra.bio).

For all analyses, the genome, transcriptome (both cDNA and ncRNA), and GTF versions used for human datasets was Ensembl GRCh38, release 98 and for mouse datasets, Ensembl GRCm38, release 99. The software versions used were as follows: *Cell Ranger* (v6.0.0), *celseq2* (v0.5.3.3), *dropSeqPipe* (v0.4.1) (YouTube tutorial link https://www.youtube.com/watch?v=4bt-azBO-18), *kallisto* (v0.46.0), *bustools* (v0.39.3), *Optimus* (v4.3.2 on Terra), *salmon* (v1.5.2 for selective alignment), *salmon* (v1.5.1) and *alevin-fry* (v0.4.0) contained in the *usefulaf* (https://github.com/COMBINE-lab/usefulaf) repository, *scPipe* (v1.8.0), *scruff* (v1.4.2), *zUMIs* (v2.5.5 on plate-based datasets and v2.9.7 on droplet-based datasets), *bowtie2* (v2.3.4.1), *samtools* (v1.9), *STAR* (v2.6.1c) and *Rsubread* (v2.0.0 for plate-based datasets and v2.4.3 for droplet-based datasets). Parameters within each preprocessing workflow were selected as recommended in the user guides. More details of each preprocessing workflow for each dataset are available at https://github.com/YOU-k/preprocess.

To compare computational performance, we created subsets of the data of varying sizes ranging from 8M to 100M for plate-based datasets and 75M to 600M for droplet-based datasets. and carried out each analysis on a high-performance cluster (Intel(R) Xeon(R) CPU E5-2690 v4 @ 2.60GHz). Maximum memory at 200GB and the time limit at 48 h were set for each submission. We required 1 node and 8 PPNs for each run and claimed 8 cores in the scripts if there were parameters that allowed this; if not, 8 threads were claimed. We ran each workflow three times on each dataset and calculated the mean and standard deviation of the performance measures, which include CPU utilization, memory usage, and run time. To measure their parallel scaling performance, threads were set as 4, 8, 12, 16, 32, and run times were recorded.

### Datasets

We selected 11 public datasets and created 1 new dataset for benchmarking (Additional file 2: Table S2). The *scmixology* datasets are available from GEO under accession number GSE118767 [5]. We selected plate-based CEL-Seq2 and 10x v2 droplet-based datasets containing cells from human lung adenocarcinoma cell lines that involved “pseudo-cells” created by mixing cells (3 datasets) or bulk RNA mixtures (1 dataset) in different combinations using CEL-seq2 or actual single cells (1 dataset with 3 cell lines and 1 dataset with 5 cell lines). Annotation files for CBs with cell types were available at https://github.com/LuyiTian/sc_mixology.

The new dataset was created using single cells from the same five human lung adenocarcinoma cell lines (HCC827, H1975, A549, H838, and H2228) that were cultured separately. Cells were counted using Chamber Slides, and roughly 2 million cells from each cell line were mixed and processed by the 10x Chromium single cell platform using v3 chemistry. Afterward, libraries were sequenced on an Illumina Nextseq 500. Raw data from this experiment are available from GEO under accession number GSE154870. To generate its annotation files, *demuxlet* (https://github.com/statgen/demuxlet) was used to deconvolve cell identity via genetic information using the intermediate bam file obtained from the *scPipe* workflow.

Another dataset profiled mouse lung tissue from the Tabula Muris study, available under GEO under accession number GSE109774 [41]. Raw bam files from channel 10X_P7_8 and 10X_P7_9 were downloaded and converted to raw FASTQ files by *bamtofastq* (v1.2.0) (https://support.10xgenomics.com/docs/bamtofastq). Annotation files for CBs with cell types were available from http://tabula-muris.ds.czbiohub.org.

The final datasets that profiled human peripheral blood mononuclear cells (PBMC) were provided by 10x. A 5k and 10k PBMC dataset were downloaded from the 10x website (from https://support.10xgenomics.com/single-cell-gene-expression/datasets/3.0.2/5k_ pbmc_v3and https://support.10xgenomics.com/single-cell-gene-expression/datasets/3.0.0/ pbmc_10k_v3). They were both sequenced with the v3 chemistry of the Chromium 10x system. To generate cell type annotation files on each dataset, we followed the online Bioconductor tutorial [69] as follows:

- Quality control of cells was performed using *scater* (v1.14.6) whereby cells with total read counts or number of genes detected greater than 3 median absolute deviations (MADs) below the median (both calculated on the log10-scale) or cells where the percentage of mitochondrial counts was 3 MADs above the median were removed from the filtered matrices downloaded from the 10x website.
- *scran* was used to normalize the data, and the top 2k highly variable genes were selected with modelGeneVar and getTopHVGs.
- The shared nearest-neighbor (SNN) graph was built using the top 20 PCs, and *cluster_louvain* was applied to cluster cells.
- A UMAP was generated on the first 20 PCs to embed the datasets into two dimensions for visualization.
- scDblFinder::findDoubletClusters (v1.4.0) was used to find clusters of doublets. Cells from doublet clusters were removed and the analysis steps after quality control were rerun.
- To assign cell types to clusters, we used canonical marker genes (Additional file 3: Figure S18). The BlueprintEncodeData [70] were also used as reference to annotate cells with the usage of *SingleR* (v1.4.1) [71].

The long-read Nanopore single-cell datasets used the same five human lung adenocarcinoma cell line mixture samples processed by the 10x Chromium single cell runs using v2 and v3 chemistry according to the protocols described in Tian et al. [49]. The long-read based count-matrices provided by the authors were used to compare the abundance of particular gene biotypes with the matching short-read data.

### Cell quality control

In an attempt to standardize cell filtering, we applied isOutlier from the *scater* package (v1.14.6) setting library size, the number of detected features, and percentage of mitochondrial genes per cell as filtering indicators with nmads = 3 for all cell-by-gene count matrices created by different preprocessing workflows. Additionally, we applied emptyDrops from the *DropletUtils* package (v1.6.1) [14] (as recommended in [69]) to all selected 10x datasets. For the lung tissue dataset and 10xv2_3cell-line dataset, *emptyDrops* failed to generate a reasonable number of cells (i.e. around 60 thousand cells in each on 10xv2_3cell-line for *zUMIs* and *kallisto bustools*, which is well above expectation of around 3k cells for other workflows), so we used *Cell Ranger* v2 filtering to distinguish true cells on these datasets. *salmon alevin* still provided relatively more cells on the 10xv2_lung-tissue1 datasets, and as most of these cells did not have a cell type label (Additional file 3: Figure S3C right-most panel). Therefore, we restricted our analysis to only include cells with labels on this dataset across workflows. Values of quality metrics after filtering are provided in Additional file 5: Table S4.

The number of detected genes per gene biotypes, total counts per gene, total counts per cell, and Pearson’s correlation were all calculated based on the cell-by-gene count matrices obtained before filtering by *scater*. Here, the correlation was calculated using common cells and common features detected across all preprocessing workflows. GLMPCs were calculated using the *GLMPCA* package (v0.2.0) and *UpSetR* (v1.4.0) plots of cells found in common between preprocessing methods after filtering with using *scater* were generated for different datasets.

Because CB annotation files were generated based on one specific workflow, and the original analyses adopted different filtering strategies, it was possible to recover cells regarded as good quality by some preprocessing workflows that were not listed in the CB annotation files. Such cells were retained for further analysis and only removed upon calculating tailored evaluation metrics that required cell labels. Doublets detected by *demuxlet* in both the CEL-Seq2 and 10x cell line datasets were removed before normalization.

### BCV plot and biological signal on raw counts

For biological noise and signals, genes with specific biotypes were firstly extracted. Next, the biological coefficient of variation (BCV) was calculated using the filtered raw count matrix using the edgeR::estimateDispersion function (v3.28.1) [72] and trended.dispersion was plotted using a loess line or directly with points. In the BCV plots, the x-axis displays the log-transformed counts obtained after *scran* normalization. Silhouette widths were calculated using the first 2 GLMPCs for cell line datasets and the first 20 GLMPCs for lung tissue and PBMC datasets.

For clustering of *n* observations (here a cluster refers to a specified group of cells), the silhouette width of observation *i* is defined as:

1 $$ sil(i) = \frac{b(i) - a(i)} {max \left(a (i), b (i) \right)} \in [{-1},1]  $$

where *a*(*i*) represents the average (here Euclidean distance based on the top selected PCs) dissimilarity between the *i*th cell and all other cells in the cluster where *i* belongs to. Here, *b*(*i*) is calculated as

2 $$ b(i) = {min}_{\mathrm{C}} d(i, C)  $$

where *d*(*i*,*C*) represents the average dissimilarity of *i* to all observations in other clusters *C*. The cluster::silhouette (v2.1.2) function [73] was used to calculate silhouette width.

To generate t_SNE plots, the same sets of genes across biotypes were extracted after normalized by *scran*. PCA was performed on them separately. The First 2 PCs on cell line datasets and the first 20 PCs on the lung tissue and PBMC datasets were used to create a t_SNE visualization.

### Data normalization

Five normalization methods were used to explore the impact the choice of preprocessing workflow has on this step and subsequent downstream analysis. The baseline *no normalization* option refers to the analysis of the raw counts directly without any further processing (this option was not used in downstream analysis). *DESeq2* (v1.26.0), *Linnorm* (v2.10.0) and *scran* (v1.14.6) were used with default settings. For *scone*, we set the maximum number of RUVg factors and maximum number of quality PCs both as 0. For *sctransform* (v0.3.2) both the default method and the method that uses *glmGamPoi* (v1.2.0) [59] were applied with 1500 features specified as the number of variable features after ranking by residual variance.

To evaluate, we performed PCA with normalized counts firstly with default settings in *scater*. Next, silhouette widths were calculated with the first 2 PCs according to known cell clusters (provided by cell line identity or mixture proportion information) for all datasets except the tissue and PBMC datasets. Considering the biological complexity of tissue and PBMC datasets, silhouette widths were calculated using the first 20 PCs. Higher silhouette widths indicated better preservation of biological signals. Additionally, variance explained by library sizes and known cell groups on the first five PCs were summed up respectively as unwanted variation and wanted variation to assess whether known biological variation was preserved and confounded technical effects were well handled on all plate-based datasets and droplet-based cell line datasets. For the tissue and PBMC datasets, variance explained from the first 20 PCs were partitioned into wanted and unwanted variation and then summed up.

### Clustering

Results from normalization were not directly applied with clustering methods. Except for *sctransform*, normalized counts were already selected with top 1.5k highly variable genes (HVGs), top 1.5k HVGs were obtained with scran::modelGeneVar and scran::getTopHVGs. Clustering methods from mainly four packages, *SC3* (v1.14.0), *Seurat* (v3.1.3), *RaceID* (v0.1.7), and *scran* with *igraph* (v1.2.5), were used. To make it easier to interpret, we provided the number of clusters or specified related parameters with a range of values to reach the true value of the number of clusters. Other parameters were specified based on either the default settings or the author’s guidance from the user manual.

For *SC3*, both the classic unsupervised method and combined Support Vector Machine (SVM) method were used. *RaceID* parameters as suggested in the user reference manual were chosen. The required number of clusters were directly provided to *SC3* and *RaceID*. For *scran*, the number of nearest neighbors was specified at 5, 10, 30, 50 and 100 to build a SNN graph. Algorithms fast greedy, Louvain, and walktrap in *igraph* were applied afterwards. For *Seurat*, we clustered specifying resolution at 0.2, 0.4, 0.6, 0.8, 1.0, 1.2, 1.4 with the algorithms of Louvain and SLM. With *RaceID* and *SC3*, the desired number of clusters can be provided directly as a parameter. When applying *scran* and *Seurat*, the clustering solution that returned either the expected number of clusters or the closest number to that were taken as the optimal solution for benchmarking.

To evaluate clustering performance, the entropy of cluster accuracy (ECA), entropy of purity (ECP) [5] and adjusted Rand index (ARI) was used to assess intra-cluster similarity, external criterion, and similarity with known clusters, respectively. ECA and ECP are defined as:

3 $$ ECA = -\frac{\sum_{i=1}^{M} \sum_{j=1}^{N_{i}} p(x_{j}) log(p(x_{j}))}{M}  $$

where *M* denotes the number of clusters generated from a method (the clustering solution to be evaluated) and *N*_*i*_ denotes in *i*th cluster based on the ground truth (here the provided labels), and

4 $$ ECP = -\frac{\sum_{i=1}^{N} \sum_{j=1}^{M_{i}} p(x_{j}) log(p(x_{j}))}{N}  $$

where *N* denotes the number of clusters from the ground truth and *M*_*i*_ denotes in *i*th cluster based on the generated clusters. ECA measures the diversity of known cell groups within each cluster provided by given methods, and ECP measures the diversity of the cluster labels within each of the known groups. Low values of ECA and ECP are favorable. The mclust::adjustedRandIndex (v5.4.7) function was used to calculate ARI.

To summarize the results of each analysis, we performed ANOVA with the following model: metric ∼ preprocess_workflow + norm_method + cluster_method + design.

We also fitted a linear model using the lm function with the listed metrics as the dependent variable and the experimental designs and specific methods as covariates. The coefficient obtained for each method indicated to what extent these methods were influencing the clustering performance. Then, the average weighted rank of coefficients on preprocessing workflows across three metrics (ARI, ECA, and ECP) are calculated as a summary. Example t-SNE plots (created using *scater*) allowed visual assessment of different combinations’ performance. The rest of the figures were created using *ggplot2* (v3.3.0) and heatmaps were created using *pheatmap* (v1.0.12).

### Benchmarking pipelines

*CellBench* (v1.2.0) was used to compare different methods as modules. The preprocessing workflows were individually applied to each dataset and the resulting cell-by-gene count matrix were input to CellBench::apply_methods() [39].

## Supplementary Information

**Additional file 1** Table S1: Summary of the scRNA-seq preprocessing workflows compared.

**Additional file 2** Information of experimental designs, GEO accession numbers, data structure, biological noise levels, and expected number of cells and number of clusters are provided.

**Additional file 3** Supplementary Figures.

**Additional file 4** Table S3: Summary of the advantages and limitations of the preprocessing workflows evaluated.

**Additional file 5** Table S4: Summary of quality control thresholds across datasets and preprocessing workflows.

**Additional file 6** Review history.

## Data Availability

The *scmixology* datasets are available from GEO under accession numbers GSE118767 [74] and GSE154870 [75]. The mouse lung tissue data from the Tabula Muris study is available under GEO under accession number GSE109774 [76]. The human PBMC datasets are available from the 10x website (the 5k dataset is from https://support.10xgenomics.com/single-cell-gene-expression/datasets/3.0.2/5k_pbmc_v3 and the 10k dataset is from https://support.10xgenomics.com/single-cell-gene-expression/datasets/3.0.0/pbmc_10k_v3). Code for the preprocessing analysis and wrappers for use in *CellBench* for the methods compared are available from GitHub at https://github.com/YOU-k/preprocess [77].
