# Supplementary material for: Benchmarking UMI-based single-cell RNA-seq preprocessing workflows
Source: Genome Biol. 2021 Dec 14;22:339. doi: 10.1186/s13059-021-02552-3 (PMC8672463; doi:10.1186/s13059-021-02552-3)
Supplement: Supplementary file 3 — Additional file 3 Supplementary Figures. [file 13059_2021_2552_MOESM3_ESM.pdf]

Supplementary Figures: Benchmarking UMI-based single cell RNA-sequencing preprocessing workflows

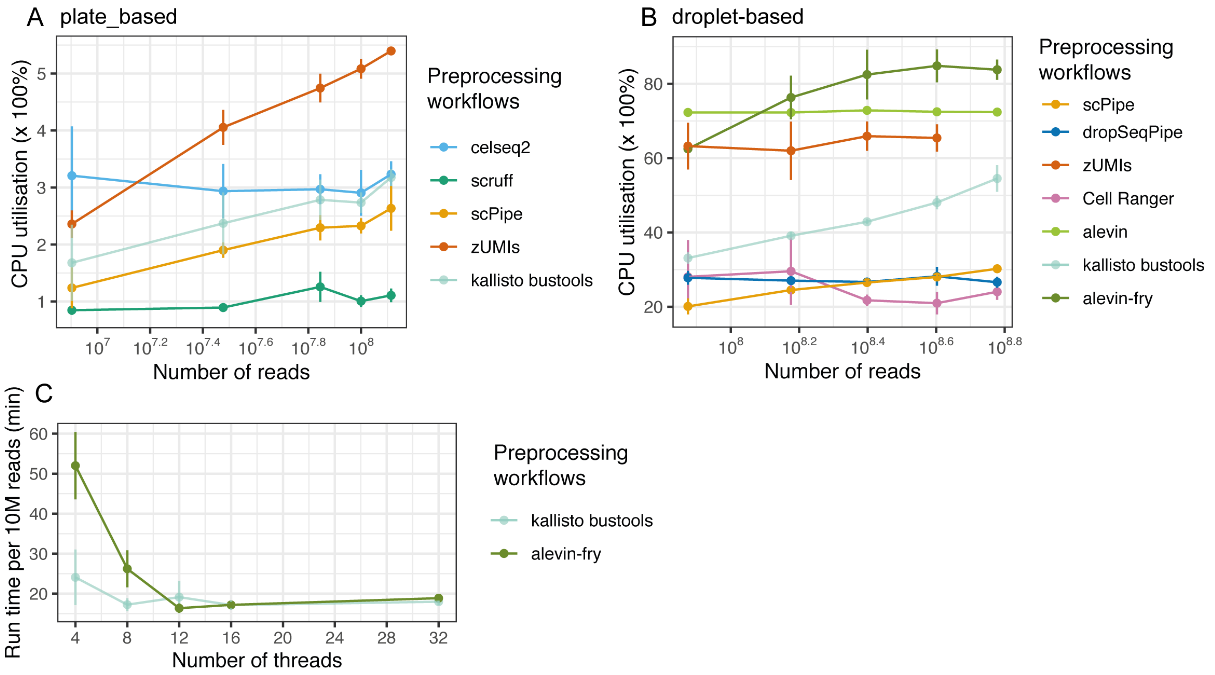

Figure S1: Comparing the computational performance of different scRNA-seq preprocessing workflows. CPU utilization for preprocessing workflows designed for **A**) plate-based protocols and **B**) droplet-based protocols are shown. Run time versus number of threads between *kallisto bustools* and *alevin fry* is shown in **C**), where run time is scaled by 10 million reads.

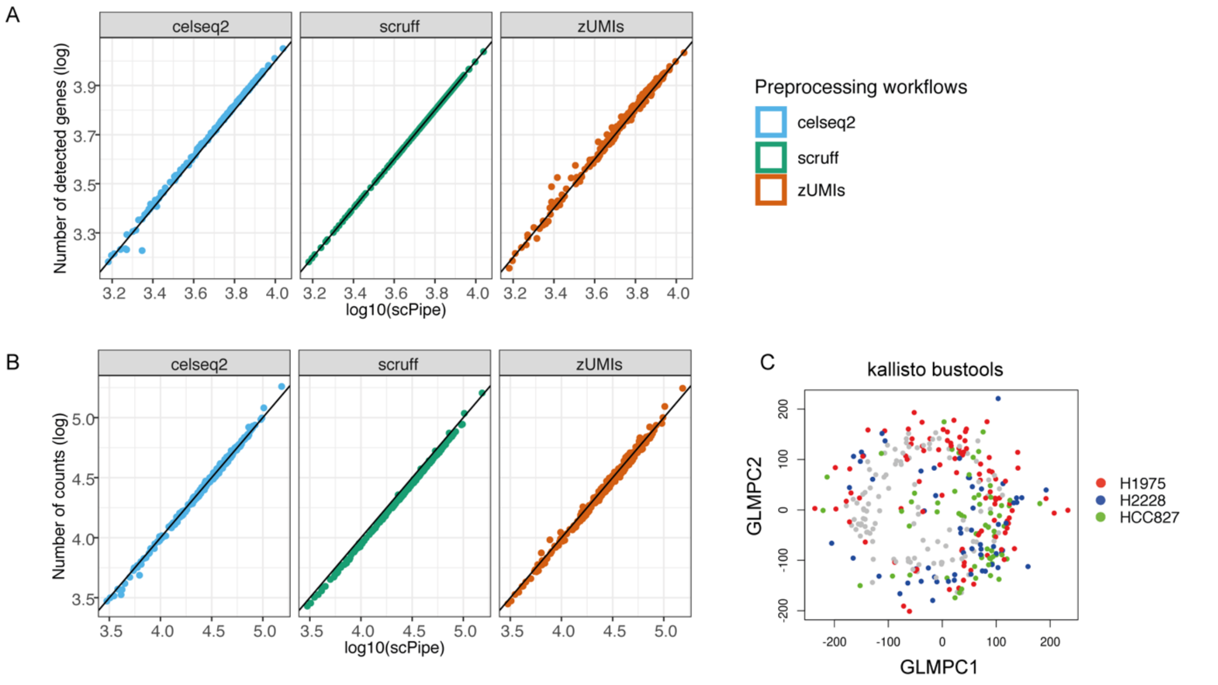

Figure S2: Comparing gene expression quantification of different scRNA-seq preprocessing workflows on the *plate_3cell-line* dataset. In terms of common cells obtained across workflows, **A**) the number of detected genes per cell and **B**) total counts per cell of different preprocessing workflows are plotted against those from *scPipe* (both in a log10-scale). GLMPCA plots delivered by *kallisto bustools* are shown in **C**). Colors represent different cell line cells. For not common cells, cells are colored in grey.

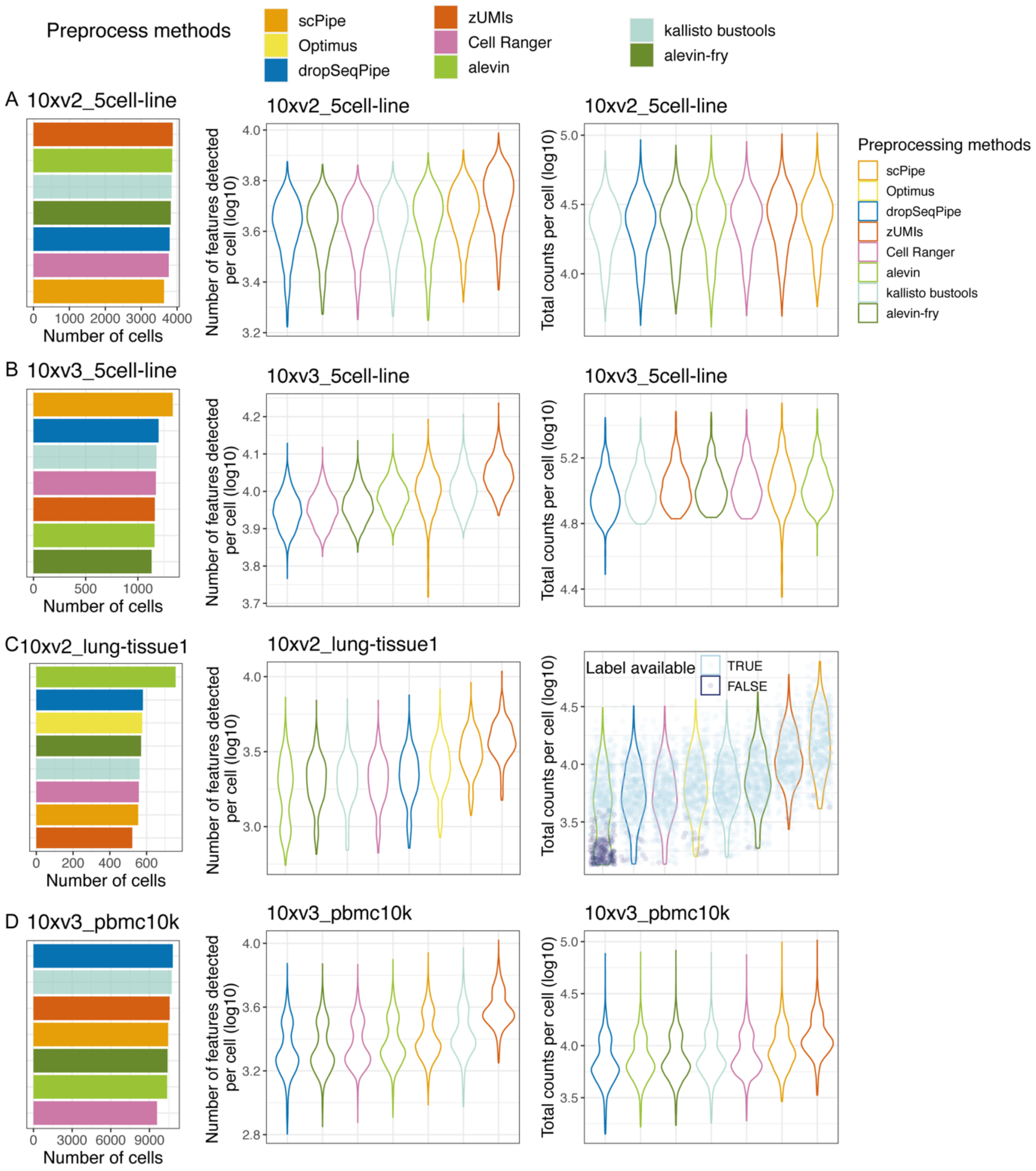

Figure S3: Number of cells, number of detected genes and total counts per cell (both in a log10-scale) on **A**) *10xv2_5cell-line*, **B**) *10xv3_5cell-line*, **C**) *10xv2_lung-tissue1*, **D**) *10xv3_pbmc10k* are shown. Color denotes preprocessing workflow. On the *10xv2_lung-tissue1* dataset (**C**, right-most panel) cells with and without labels are plotted in different shades of blue.

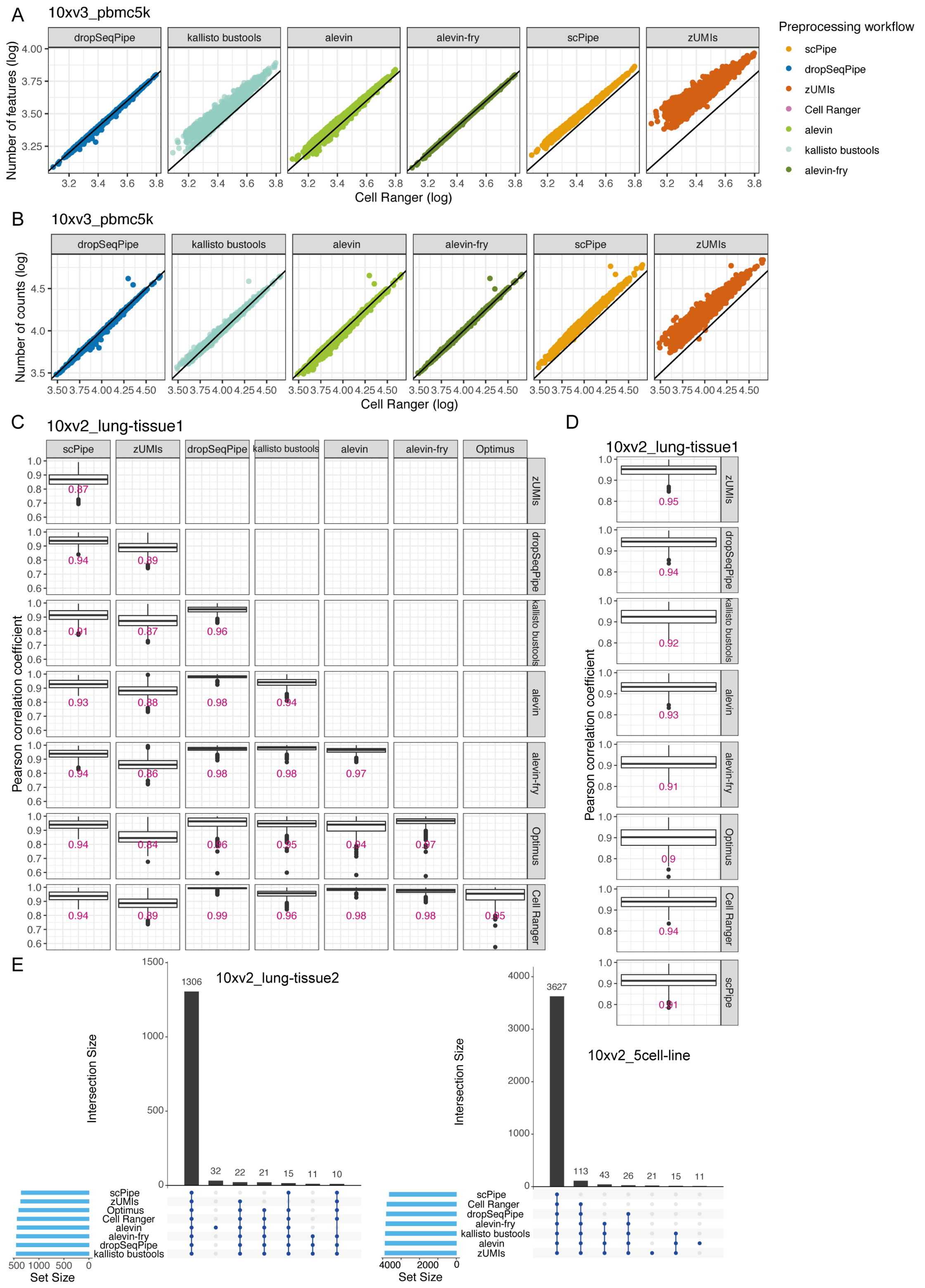

Figure S4: Number of detected genes per cell and total counts per cell of common cells are plotted of listed preprocessing workflows against *Cell Ranger* accordingly on *10xv3_pbmc5k* in **A**) and **B**) (all on the $\log_{10}$-scale). Pearson correlation coefficients are calculated using common genes in individual cells across selected preprocessing workflows on *10xv2_lung-tissue1* datasets, and then plotted in **C**). The median value of the Pearson correlation coefficient is labelled in pink in each boxplot. Additionally, Pearson correlation coefficients of *zUMIs* run in exon mode with other workflows are shown in **D**). The UpSet plots in **E**) are used to display intersections of retains cells across workflows on *10xv2_lung-tissue2*, and *10xv2_5cell-line* datasets.

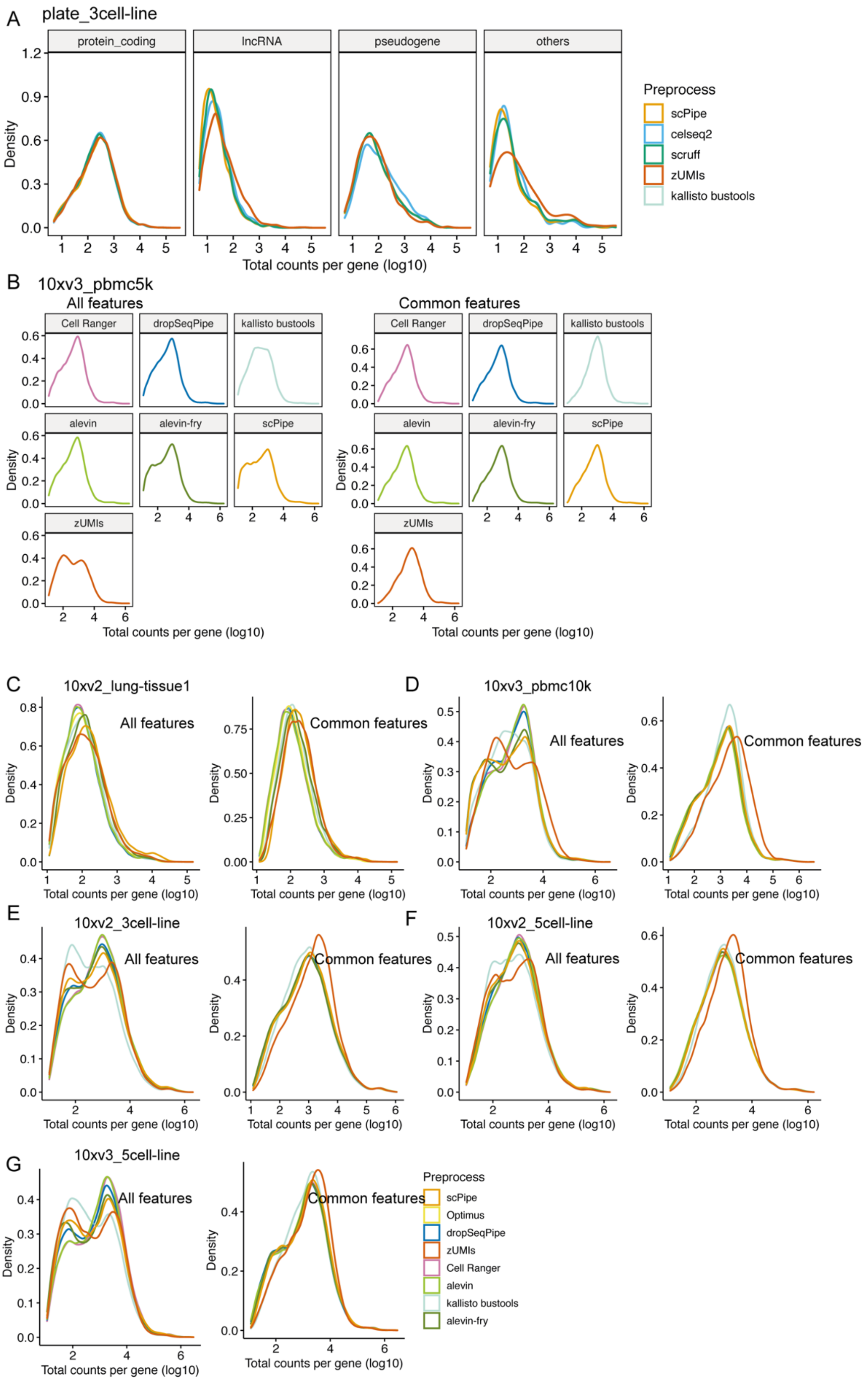

Figure S5: Density plot of total counts per gene, facet by gene biotypes on *plate_3cell-line* dataset is shown in A). For *10xv3_pbmc5k* dataset, density of total counts per gene, faceted by gene biotypes and preprocessing workflows are shown in **B**) to avoid overplotting. For other droplet-based datasets, the density of total counts per gene of all features and common features across workflows (on a $\log_{10}$-scale) are shown in **C**) *10xv2_lung-tissue1*, **D**) *10xv3_pbmc10k*, **E**) *10xv2_3cell-line*, **F**) *10xv2_5cell-line* and **G**) *10xv3_5cell-line* datasets.

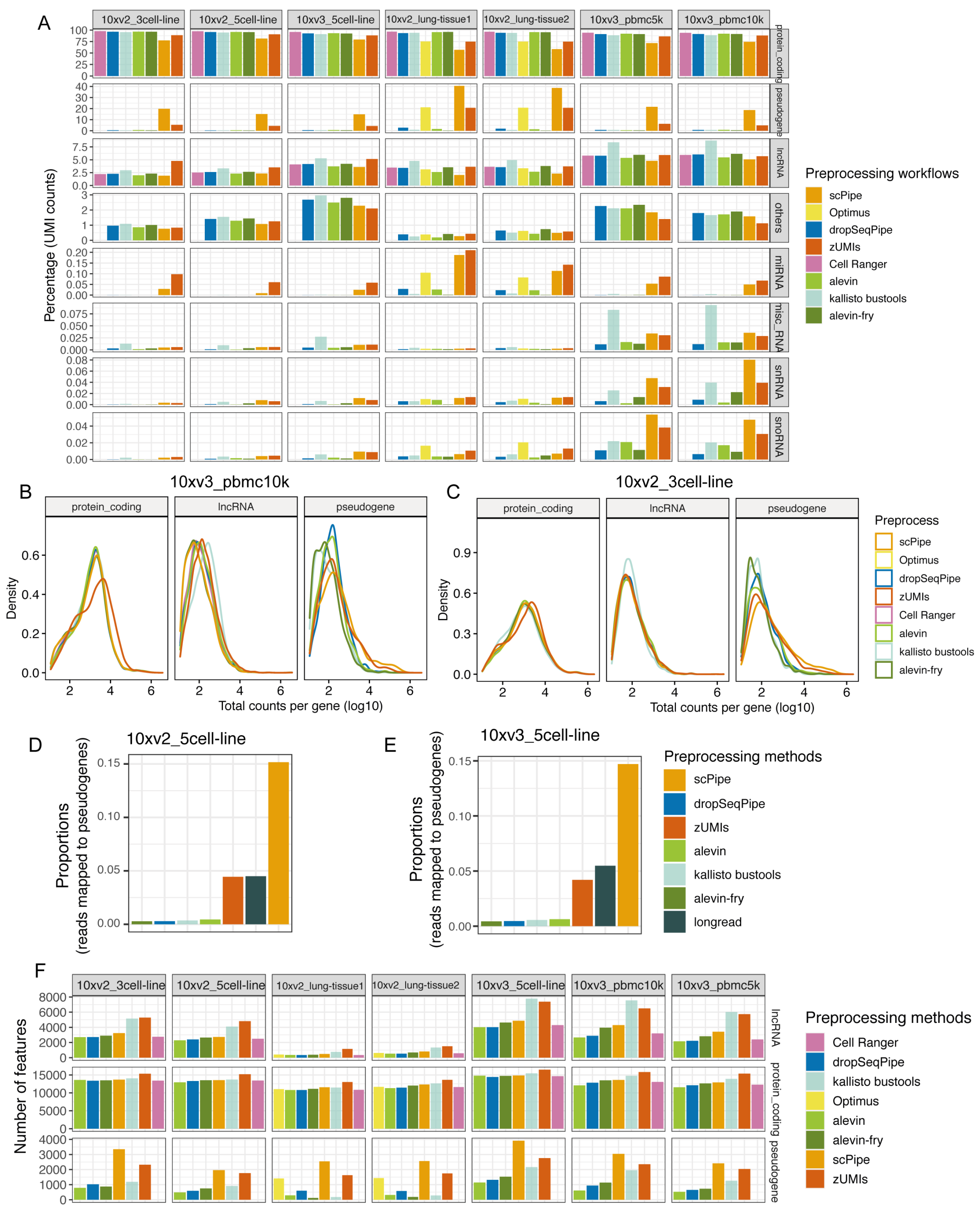

Figure S6: A) Bar plots are used to show proportions of genes of listed gene biotypes delivered across different preprocessing workflows on droplet-based datasets. Gene biotypes of Long non-coding RNAs (lncRNAs), microRNAs (miRNAs), Miscellaneous RNAs (misc-RNAs), protein coding genes, pseudogenes, small nuclear RNAs (snoRNAs), and small nucleolar RNAs (snRNAs) are shown here. Colors represent preprocessing workflows. The density of total counts per gene facet by gene biotypes on *10xv3_pbmc10k* **B**) and **C**) *10xv2_3cell-line* datasets are shown. Comparisons of proportions of counts mapped to pseudogenes across selected workflows on short-read sequencing and that obtained from single cell long-read sequencing on **D**) 10xv2_5cell-line dataset and **E**) *10xv3_5cell-line* dataset. Color denotes preprocessing workflows and results from long read data is colored by dark grey. **F**) Number of features of listed biotypes delivered by different preprocessing workflows on across datasets. Only genes of lncRNA, protein coding genes, and pseudogenes were extracted from the raw count matrices and then used in the following evaluation.

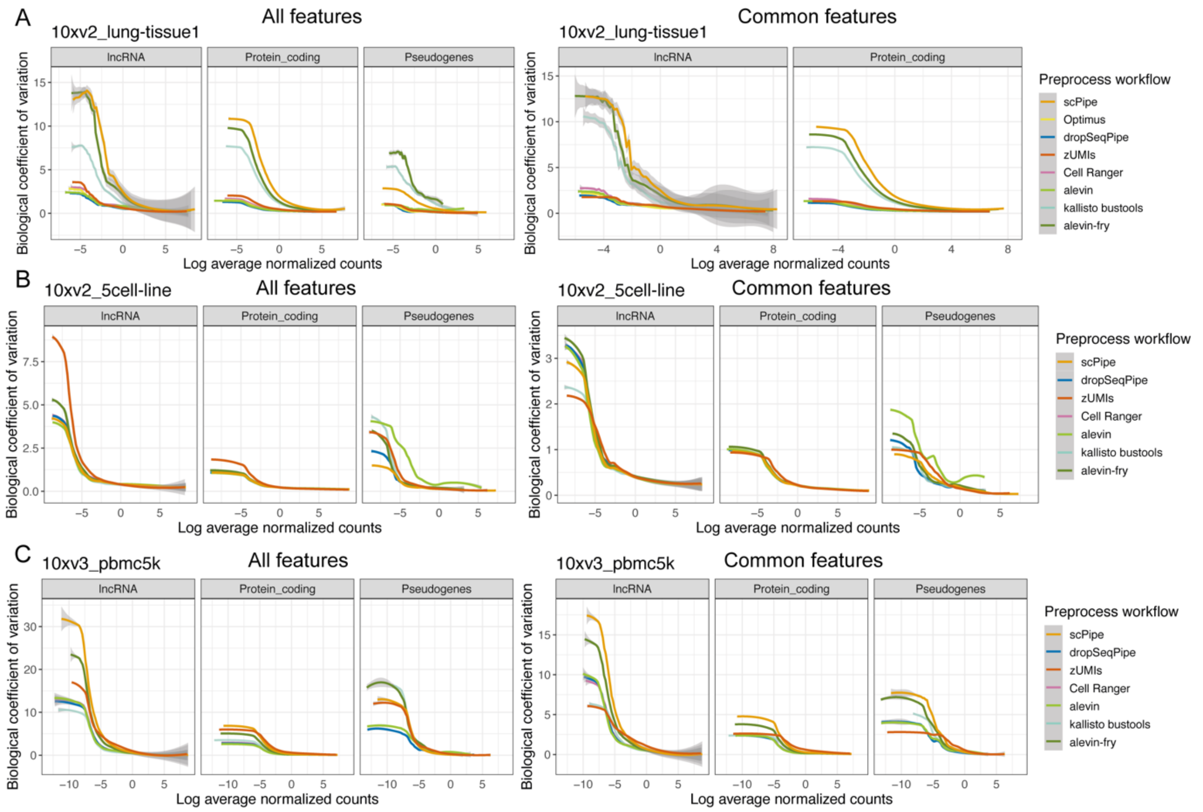

Figure S7: BCV plots of genes with biotypes of lncRNA, protein coding genes and pseudogenes delivered by different preprocessing workflows on **A**) *10xv2_lung-tissue1*, **B**) *10xv2_5cell-line* and **C**) *10xv3_pbmc5k* datasets using all features and common features across workflows accordingly. Raw gene counts from features of different biotypes were used to calculate biological coefficient of variation (BCV), with BCV values are plotted against *scran* normalized average counts (log-scale). Colors represent preprocessing workflows.

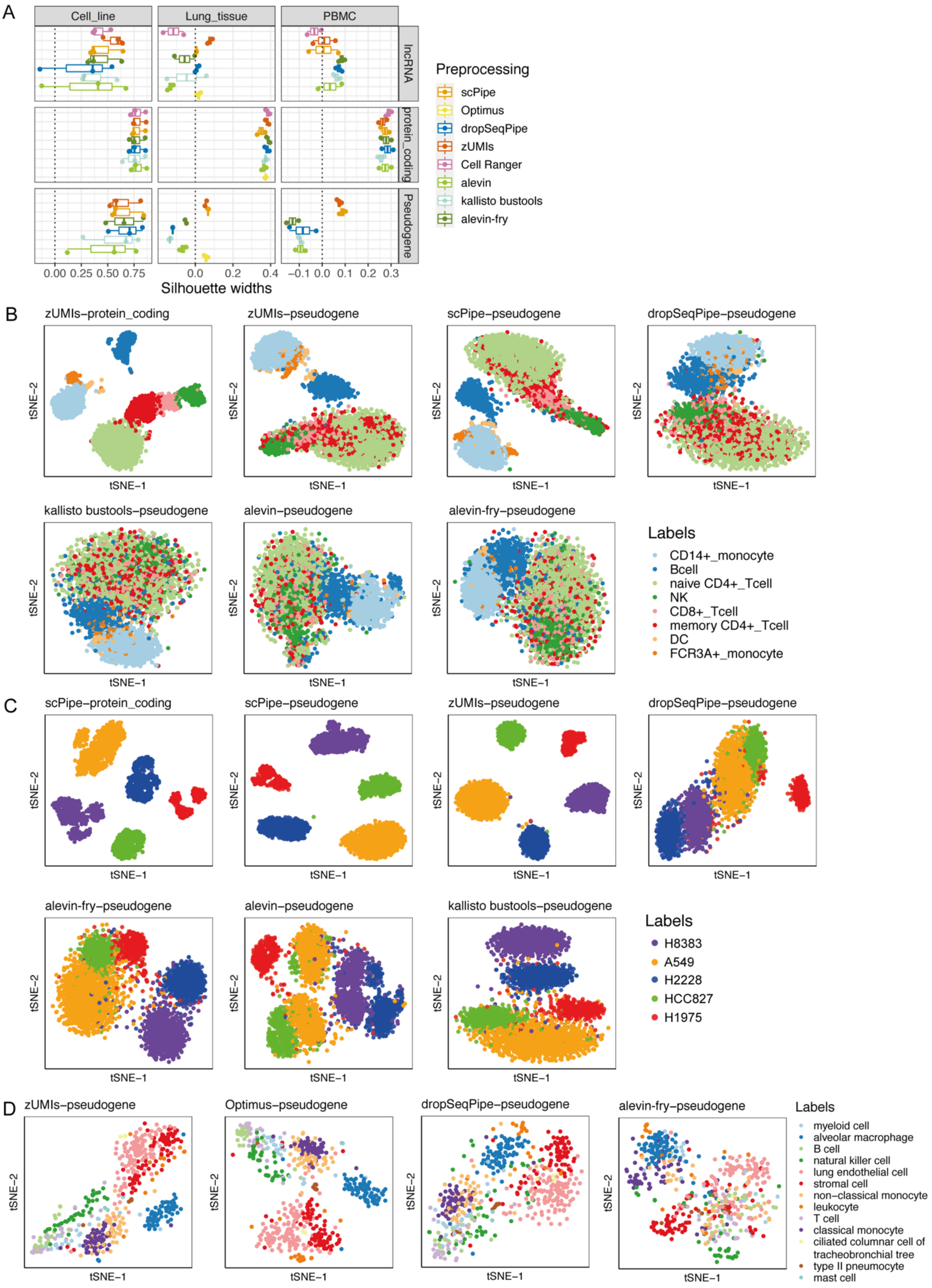

Figure S8: Boxplots of silhouette widths calculated with GLMPCs obtained from different workflows based on known cell types are shown in **A**) faceted by different experimental designs. Silhouette width=0 is shown with a black dashed line. t-SNE plots generated with genes of specific gene biotypes on **B**) *10xv3_pbmc5k*, **C**) *10xv2_5cell-line* and **D**) *10xv2_tissue1* datasets. Protein coding genes, lncRNAs, and pseudogenes are extracted from *scran* normalized counts and then visualized with t-SNE plots. Colors denotes the cell type labels.

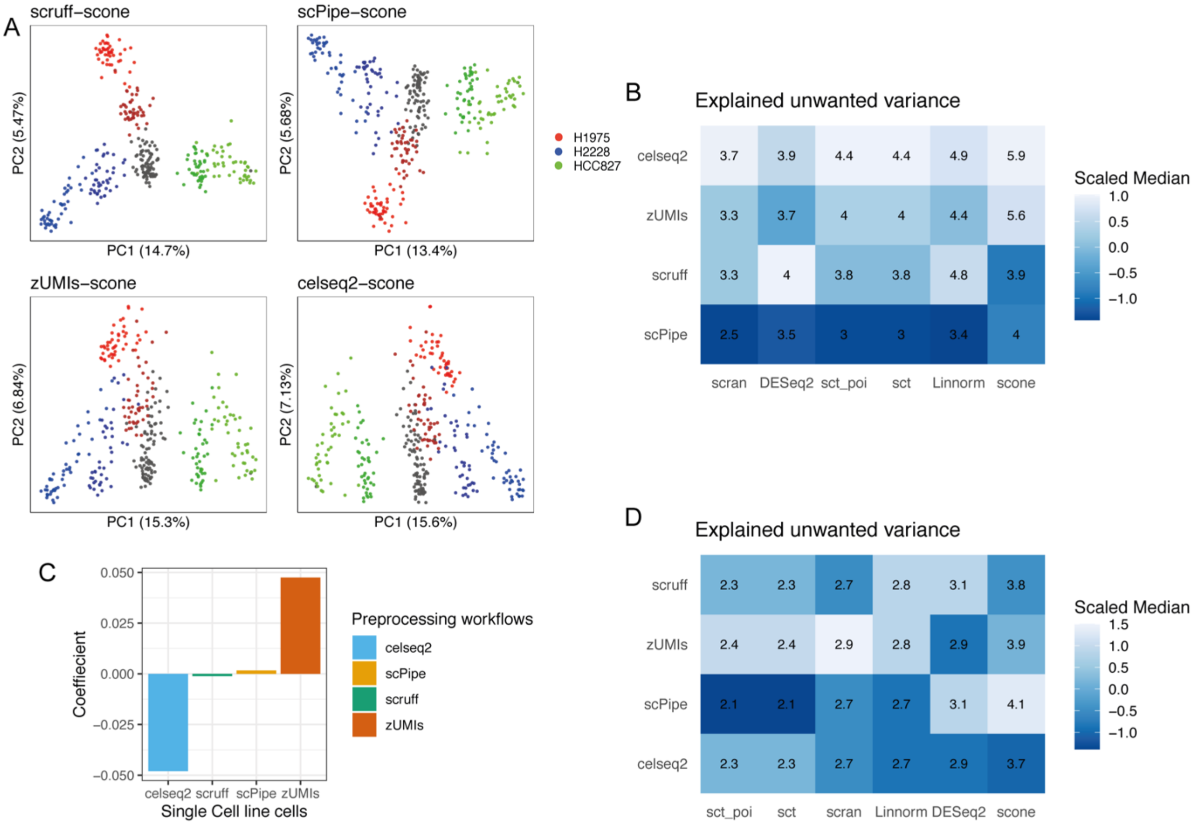

Figure S9: PCA plots for different combinations of preprocessing workflows and normalization methods applied to the *RNA mixture* data are displayed in **A**). Heatmaps of median values of unwanted variance calculated across different preprocessing workflows are shown for the RNA mixture (**B**) and cell line datasets (**D**). *sct* represents *sctransform* and *sct_poi* represents *sctransform* with *glmGamPoi*. On the plate-based cell line datasets, a linear model is fitted using silhouette widths as dependent variables, with preprocessing workflows as covariates, with the resulting coefficients plotted in **C**).

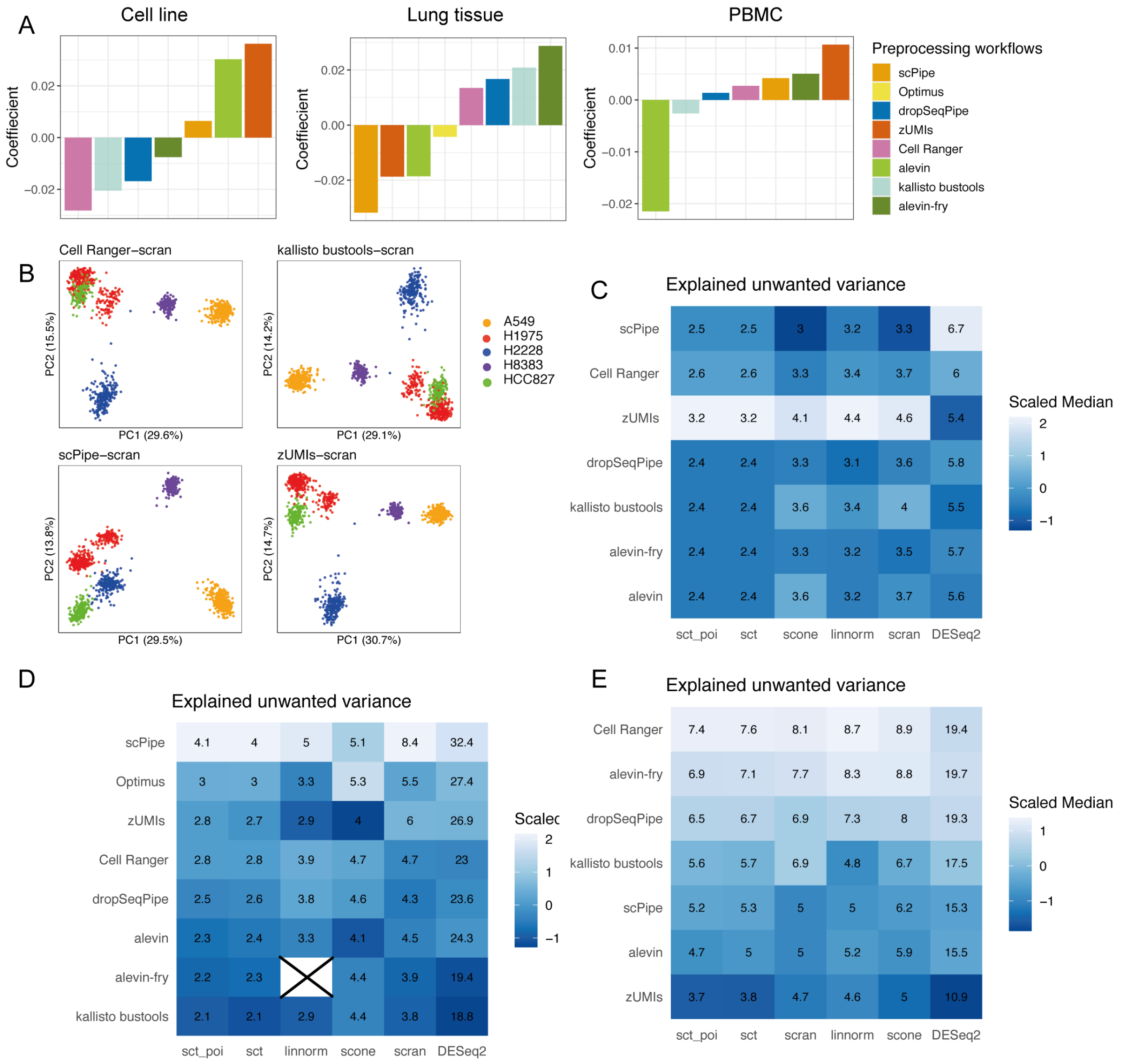

Figure S10: Linear models are fitted using silhouette widths as dependent variables, with preprocessing workflows as covariates using combinations obtained after normalization on datasets with different experimental designs. Coefficients are plotted in **A**). PCA plots based on *scran* normalized counts from different preprocessing workflows run on the *10xv3_5celline* dataset are displayed in **B**). *sct* represents *sctransform*. *sct_poi* represents *sctransform* run with the *glmGamPoi* method. Heatmaps of median values of explained unwanted variance calculated with different preprocessing workflows on the *10xv2_3celline* (**C**), *10xv2_5cell-line* (**D**) and *10xv3_5cell-line* (**E**) are shown.

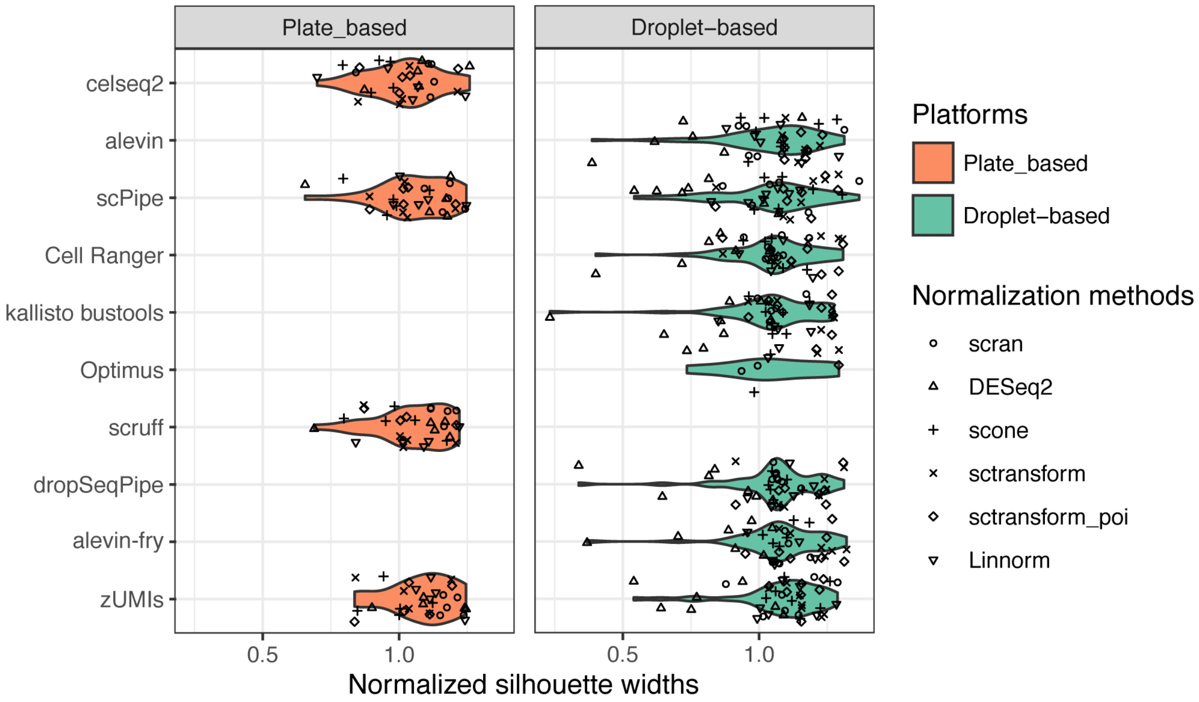

Figure S11: Summary of performance of different combinations of preprocessing workflows and normalization methods. From top to bottom, the performance is ordered from the lowest to highest median normalized silhouette widths. Silhouette widths are calculated based on known cell labels after applying different normalization methods and normalized against the silhouette widths obtained without any normalization. Here, each dot represents a combination. Colors denote different single cell platforms and shapes denote different normalization methods.

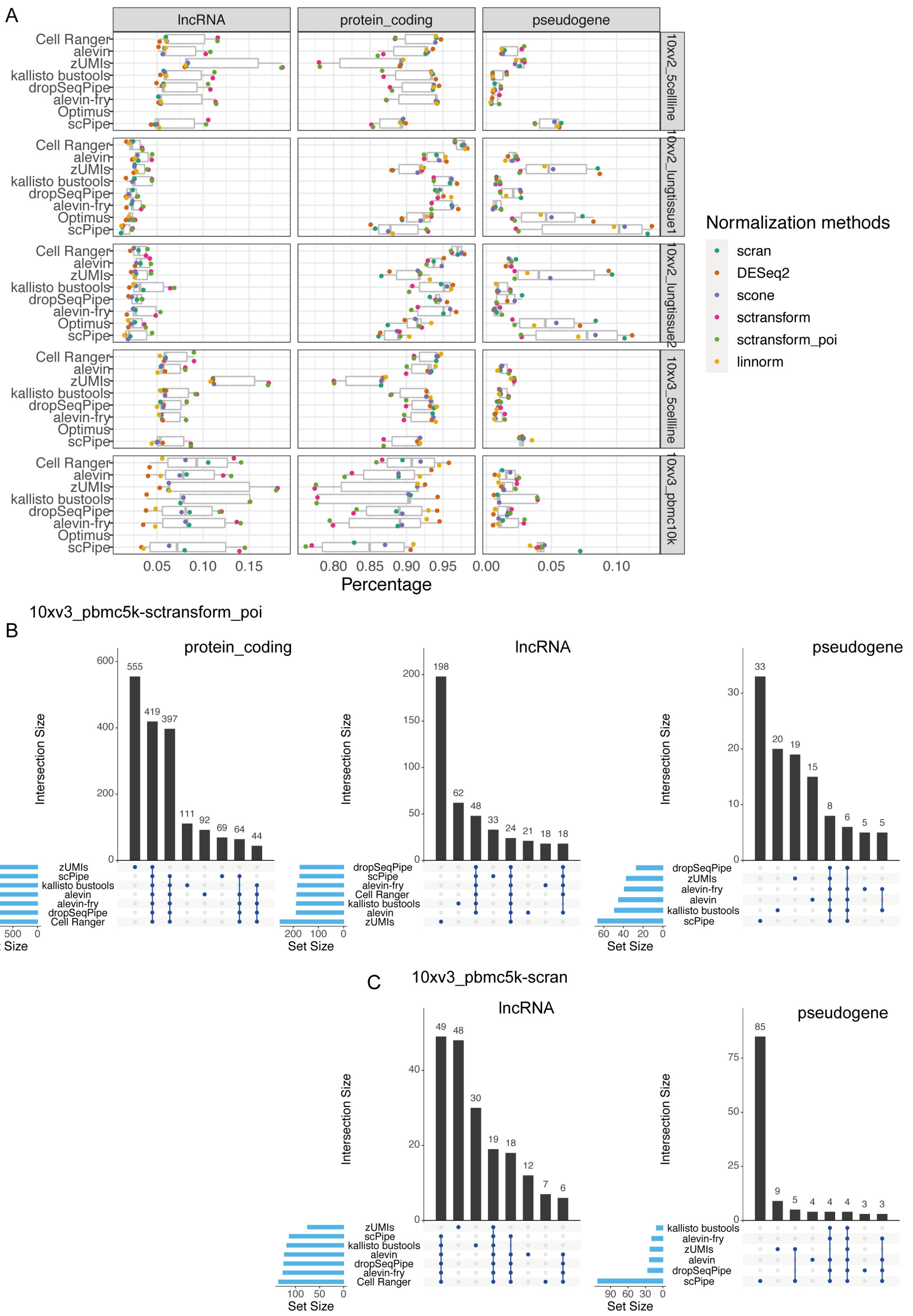

Figure S12: Percentages of different gene biotypes, including lncRNAs, protein coding genes, and pseudogenes in HVGs across droplet-based datasets are plotted in **A**). Color denotes normalization method.

Take results normalized by **B**) *sctransform* run with the *glmGamPoi* method and **C**) *scran* as examples, intersections across preprocessing workflows of top 1.5k HVGs split by listed gene biotypes on *10xv3_pbmc5k* datasets are shown using UpSet plots.

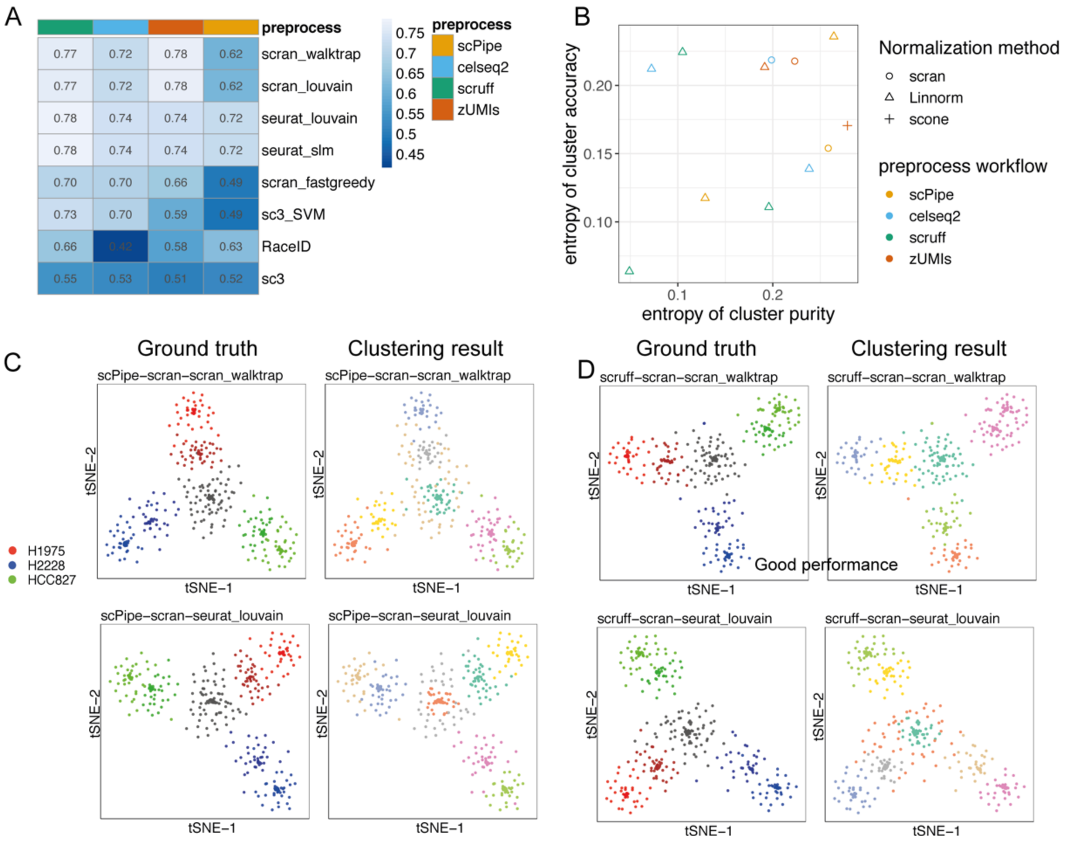

Figure S13: Comparing the impact preprocessing workflows have on clustering results on plate-based *RNA mixture* datasets. Heatmap of the median values of ARI is shown in **A**). ECA versus ECP plot for top 3 combinations delivered by different preprocessing workflows is shown in **B**). Color denotes preprocessing workflows, and shape denotes normalization methods. Example t-SNE plots generated with different combinations of preprocessing workflows, normalization and clustering methods are in **C**) and **D**). For each combination, two t-SNE plots are shown. The left t-SNE plot is colored by ground truth (known cell labels), and the right t-SNE plot is colored by the clusters identified.

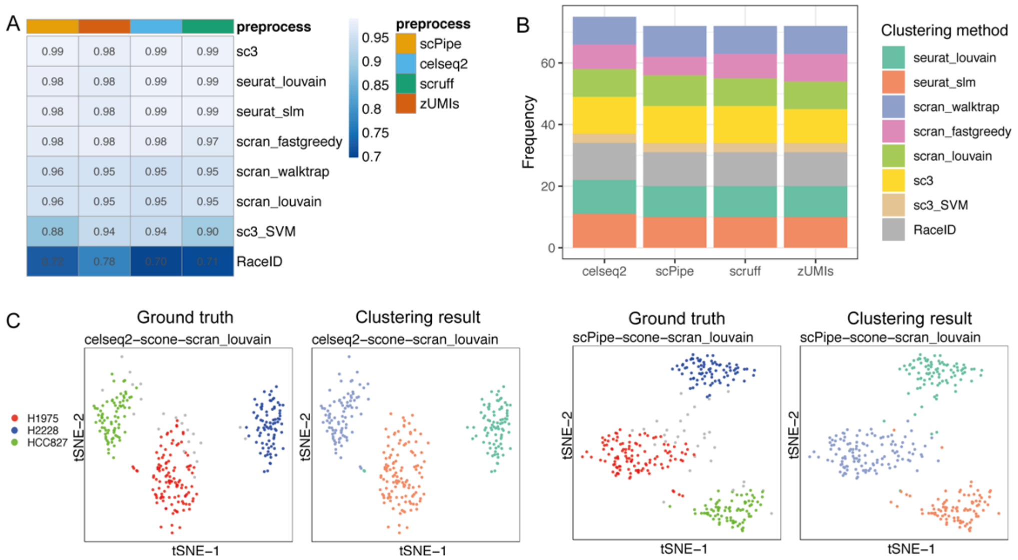

Figure S14: Comparing the impact preprocessing workflows have on clustering results on plate-based cell line cells datasets. Heatmap of the median values of ARI is shown in **A**). **B**) Number of combinations reached both ECA=0 and ECP=0 across preprocessing workflows. Example t-SNE plots generated with different combinations of preprocessing, normalization and clustering methods are shown in **C**). For each combination, two t-SNE plots are presented. The left t-SNE plot is colored by ground truth (known cell labels), and the right t-SNE plot is colored by the clusters identified.

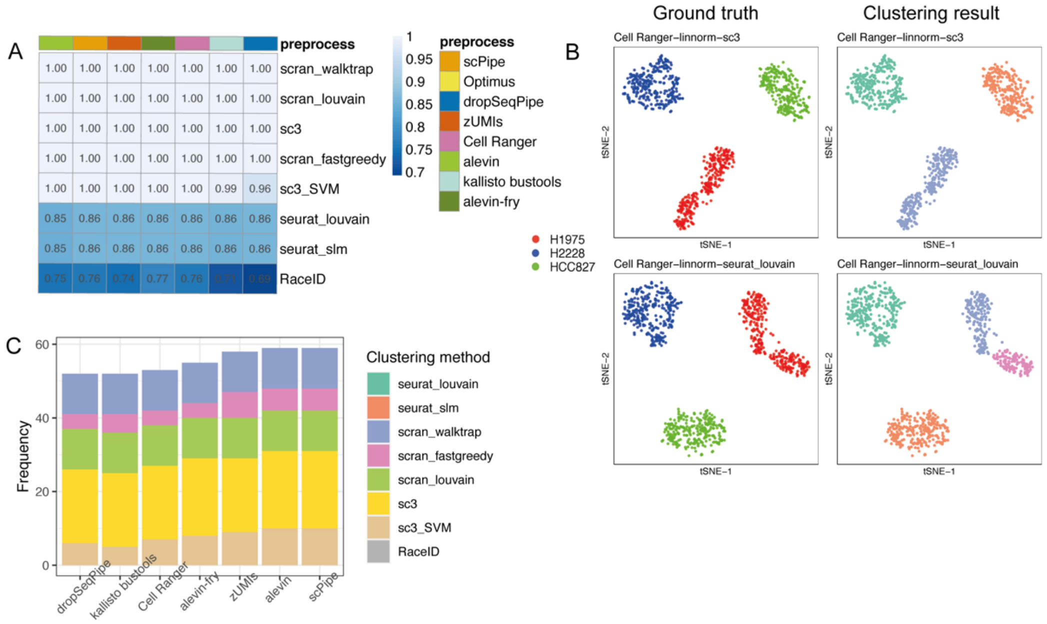

Figure S15: Comparing the impact preprocessing workflows have on clustering results on droplet-based cell line cells datasets. Heatmap of the median values of ARI is shown in **A**). Example t-SNE plots generated with different combinations of preprocessing, normalization, clustering methods are in **B**). For each combination, two t-SNE plots are shown. The left t-SNE plot is colored by ground truth (known cell labels), and the right t-SNE plot is colored by the cluster identified. **C**) Number of combinations that reached both ECA=0 and ECP=0 across preprocessing workflows.

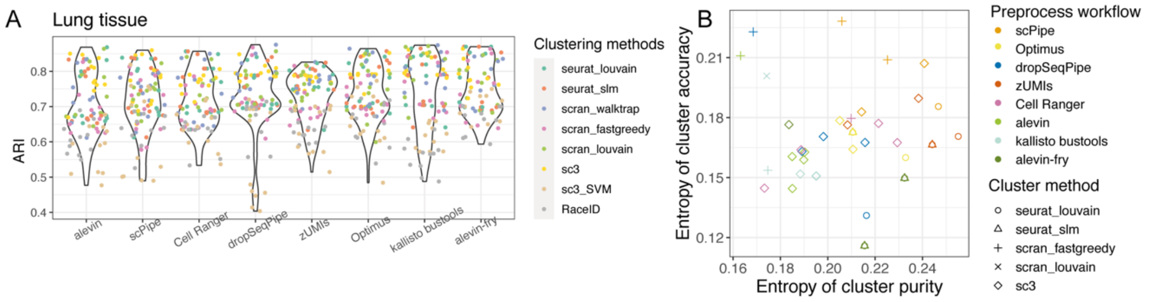

Figure S16: Comparing the impact preprocessing workflows have on clustering results on droplet-based lung tissue cells datasets. **A**) Performance evaluated by ARI is displayed in violin plots broken down by different preprocessing workflows. Each dot represents a combination and is colored by the clustering method applied. ECA versus ECP plot for top 5 combinations delivered by different preprocessing workflows is shown in **B**). Color denotes preprocessing workflows, and shape denotes clustering methods.

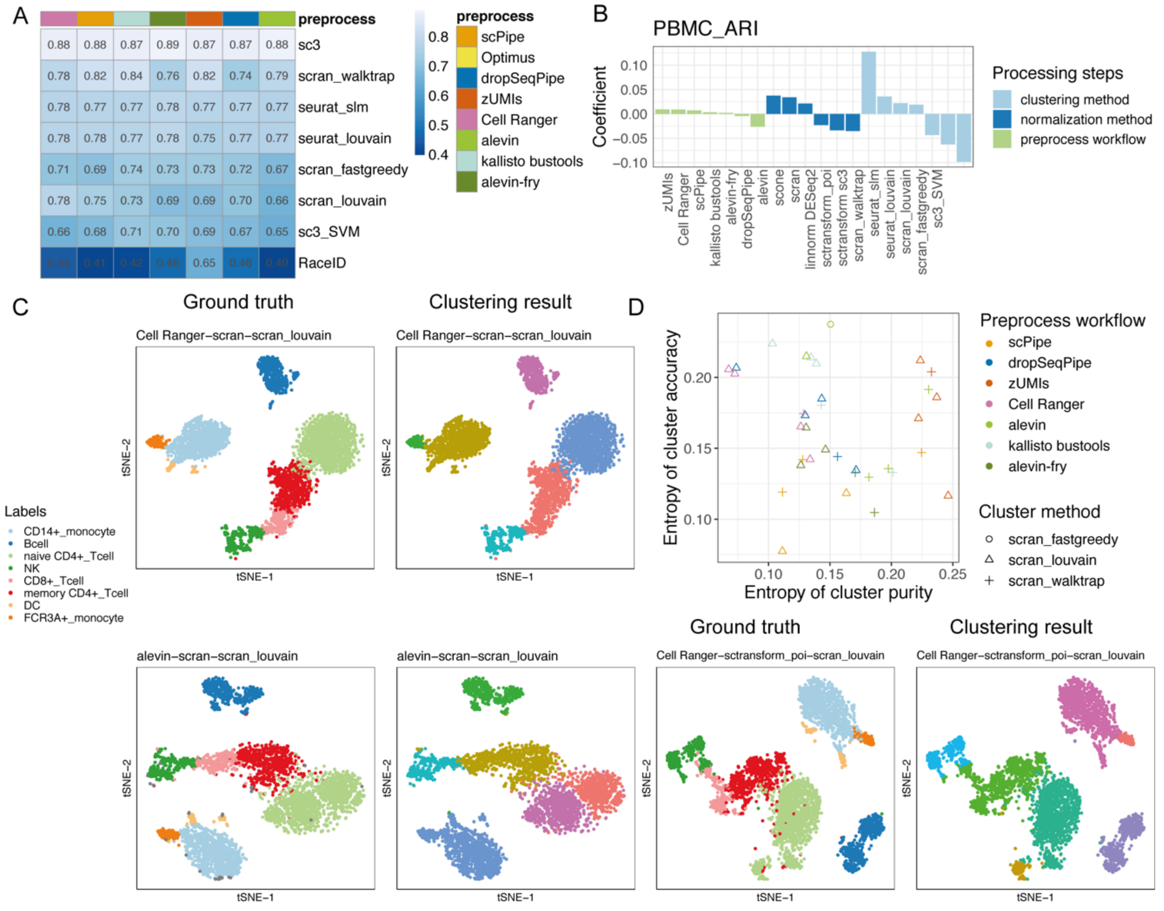

Figure S17: Comparing the impact preprocessing workflows have on clustering results on droplet-based PBMC datasets. Heatmap of the median values of ARI is shown in **A**). A linear model is fitted using ARI as dependent variables, with preprocessing workflows, normalization methods, and clustering methods as covariates. Coefficients are plotted in **B**). Example t-SNE plots generated with different combinations of preprocessing, normalization and clustering methods are shown in **C**). For each combination, two t-SNE plots are presented. The left t-SNE plot is colored by ground truth (known cell labels), and the right t-SNE plot is colored by identified clusters. ECA versus ECP plot for top 5 performing combinations from each preprocessing workflow is shown in **D**). Color denotes preprocessing workflow, and shape denotes clustering method.

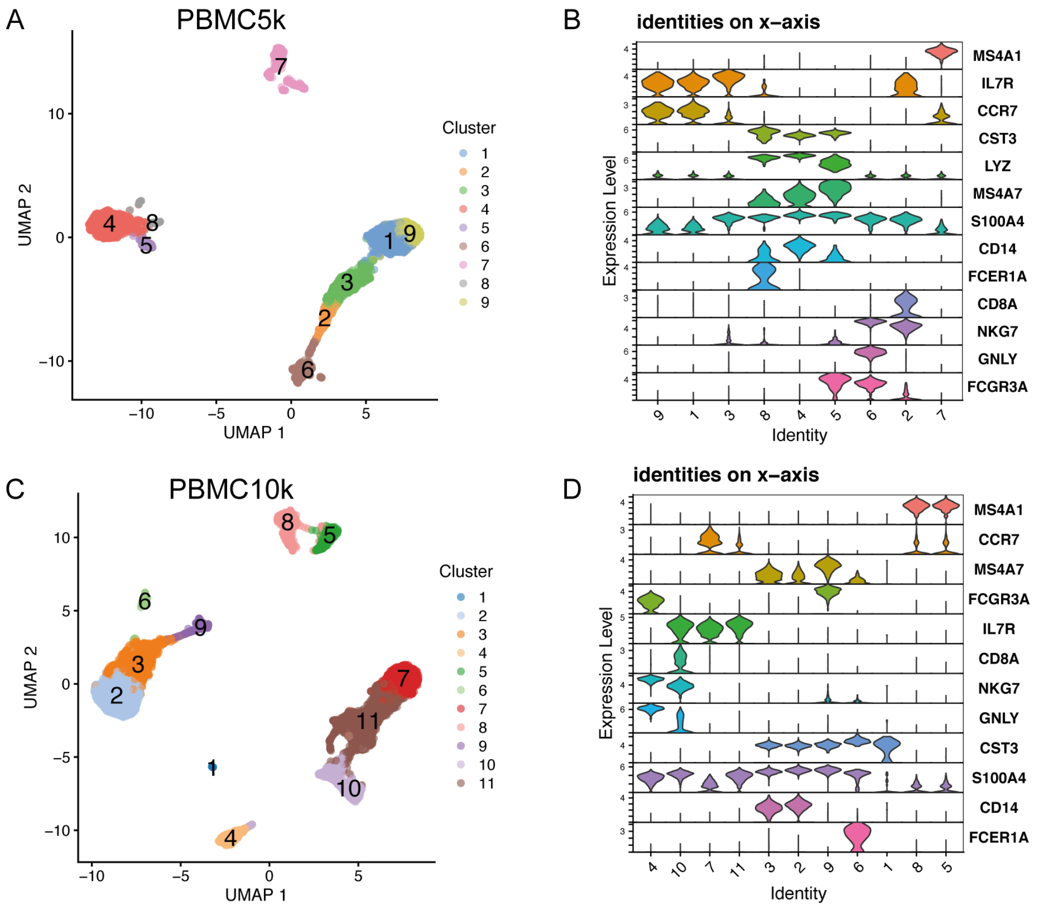

Figure S18: UMAP plots of clustering results on **A**) 10xv3_pbmc5k and **C**) 10xv3_pbmc10k datasets. Color denotes idenitified clusters. To annotate these clusters, violin plots of canonical immune markers are shown in **B**) on the 10xv3_pbmc5k and **D**) on the 10xv3_pbmc10k datasets.
