# Supplementary material for: Benchmarking UMI-based single-cell RNA-seq preprocessing workflows
Source: Genome Biol. 2021 Dec 14;22:339. doi: 10.1186/s13059-021-02552-3 (PMC8672463; doi:10.1186/s13059-021-02552-3)
Supplement: Supplementary file 6 — Additional file 6 Review history. [file 13059_2021_2552_MOESM6_ESM.docx]

Review History

**First round of review**

**Reviewer 1**

**Are you able to assess all statistics in the manuscript, including the appropriateness of statistical tests used?**

Yes, and I have assessed the statistics in my report.

**Comments to author:**

The manuscript presented by Yue You and colleagues proposes a benchmarking study of the performance of 9 different preprocessing workflows (Cell Ranger, Optimus, salmon alevin, kallisto bustools, dropSeqPipe, scPipe, zUMIs, celseq2 and scruff) for scRNAseq data.

They tested these methods on datasets originated from CEL-seq2 (plate-based) and 10X Chromium (droplet-based) technologies. They focused their analytical results on compu- tational performance, gene quantification, and gene biotype detection, along with perfor- mance assessment in downstream analyses, more specifically in normalization methods, identification of highly variable genes (HVGs) and cell clustering. They claim they have benchmarked more than 2,000 pipelines, results of combining different datasets, prepro- cessing workflows and downstream analysis methods. Their findings demonstrate that the selection of preprocessing workflow does not carry a big impact on scRNAseq analy- sis, with relatively minor differences among these combinations. These differences could even change with the most up-to-date versions of the selected softwares (i.e. Cell Ranger version 6.0.0 instead of v3.0.2).

The study motivation and importance, the standard steps of a preprocessing workflow, some differences among the selected softwares, the current state of research and limita- tions of previous benchmarking studies are clearly and orderly stated in the introductory "background" section of the paper. Because the topic discussed in this paper revolves around UMI-based methods, UMIs themselves and their role in scRNAseq could be fur- ther described in the paragraphs where they are first mentioned (p.2, lines 14-16).

The overall read of the results section feels lengthy and dense (11 pages), and the dis- cussion could be shortened and made more concise. It is not clear the choice of main text figures and supplementary figures, as in some sections the supplementary figures are referenced more often and seem more important than the "main" ones (For example p.13 lines 6-36). This happens again in the clustering section, where no tSNE or UMAPs plots are shown as main figures but rather kept as supplementary and referenced broadly all over the text. Throughout this section, the authors document several discrepancies between their results and other previous studies (p.5 line 48, p.6 lines 6-8, p.10, lines 43-46), for which they offer vague hypothetical reasons to explain why.

Chosen datasets are not representative of common scRNAseq datasets used nowadays. They do not portray big cellular heterogeneity, and more real sequenced tissues with annotated cells should have been compared. When assessing computational resources, the largest total number of reads is only 108, which falls short for most widely used datasets nowadays. This is admitted in the Discussion section (p.17, lines 20-24).

Suggestions:

* In the methods section (page 19), they could provide a table indicating the QC threshold applied to each of the datasets, allowing reproducibility.

* The names of "single cell dataset" and "tissue dataset" are confusing, since both are composed of single cell experiments.

* The metric silhouette width is not explained, while other metrics used here for clustering assessment (ECA, ECP and ARI) are. Also, should their formulas be shown in the Methods section?

* I've personally found hard to compare values between figures S15A and S12A, due to the design of these tile plots.

* The tile plots in Fig.7 (7C and 7D) should contain rank values, so the reader can make not only a qualitative comparison depending on the color but also a quantitative one.

* Table 1 is oversimplistic and vague. For example, it is not explained what con- stitutes a "User friendly" software, when a YouTube tutorial for dropSeqPipe is mentioned but not the whole amount of documentation and tutorial behind Cell Ranger webpage.

Errors and typos:

p.6 lines 33-35: The sentence is weirdly written.

p.11 line 61: They're referencing figure S8B, not S7B.

p.14 line 34: onserved instead of conserved.

p.17 line 8: repeated to particle.

p.20 lines 31-34: is weirdly written.

p.20 line 52: the middle sentence is bad written.

p.20 line 62: the last sentence is bad written.

p.21 line 17-18: Which "other figures"? This is very vague.

**Reviewer 2**

**Are you able to assess all statistics in the manuscript, including the appropriateness of statistical tests used?**

Yes, and I have assessed the statistics in my report.

**Comments to author:**

In this manuscript authors have benchmarked various preprocessing workflows for UMI based single-cell RNA sequencing technologies. Overall the manuscript is excellently written and provide important insights onto which methods are important for specific use cases. I have a few minor comments which can improve the clarity and impact of the paper to the broader community:

Comments:

* The motivation to use CEL-seq1/2 technology for benchmarking should be clarified, currently the motivation to benchmark with only CEL-seq for well based method is unclear.

* It's unclear why authors chose to not benchmark alevin on CEL-seq data, especially when they mention in Page 3 Line 60, that alevin can quantify CEL-seq data.

* Figure1: Salmon alevin can perform both pseudoalignment and end-to-end alignment, hence I am unclear about putting alevin into the category of pseudoaligners.

* Figure2: As more recent single-cell technologies can profile millions of cells it's worth checking how computational tools scales when given more resources. Henceforth it'd be worth comparing the scalability of the tools wrt number of threads.

* Figure 3F I It's unclear why is there no kallisto based umap ? I know authors chose to exclude kallisto from benchmark but having the umap based on kallisto quantified data makes the case even stronger, if it's not separating the cells based on cell-lines.

* On Page 8 Line 9: Authors mention "Calculating correlations with intron counts ex- cluded by zUMIs didn't noticeably increase the correlation values" but from Figure S3F, it seems the overall correlation seems to increase, no ?

* Figure 4A (10xv2_tissue2) The results from alevin pipeline is unclear as the number of cells are significantly high. It's worth checking the command line flags used for quantifying the data, specially `--expectCells` flag which can be provided to improve the number of cells, as also provided to the Cellranger pipeline.

* Figure 5(B-C, E-H): Since most of the lines in the figures overlap, it's hard to interpret it. Also the point of having solid lines versus dotted lines is unclear. I think the figure can be interpreted better with disjoint plots of each tool, like Violin Plots.

* Color coding of Fig S4D is not clear, for example what is the annotation of the pink color ?

* Page 10 line 52, Authors mention "These results suggest that focusing analysis efforts on the signal from protein coding genes in datasets that profile complex tissues with greater cell type diversity may be an optimal strategy." Are they talking about pseudoalignment methods specifically or in general for all other methods ?

* Page 11 line 61: Authors mention PCA plots on Fig7B, but it seems they are Violin plots, I am guessing this one is a typo.

Optional Comments: Based on authors discretion they can chose or not to perform the analyses, but it can improve overall impact of the paper:

* Multimodal data benchmarking: Advent of multimodal technologies have enabled measurement of multiple single-cell modalities within a single-cell. For example CITE-seq can profile both cell-surface proteins and RNA-seq on a droplet based single-cell system. The biggest advantage of these methods is the availability of robust protein measurements methods, which can be used to cluster cells and the second modality such as RNA-seq can be validated against. It'd be interesting to know how different scRNA-seq preprocessing workflows trades off compared to a clustering based on protein data.

* New generation of methods: Recently the authors of SCtransform and alevin have released newer version of their software: SCtransform 2 (Satija Lab) and alevin-fry (Patro Lab). Although both the methods are not yet peer-reviewed (only preprinted), it'd still be interesting to know how these methods stand in the current benchmarks.

**Authors Response**

**Point-by-point responses to the reviewers’ comments:**

Reviewer #1

The manuscript presented by Yue You and colleagues proposes a benchmarking study of the performance of 9 different preprocessing workflows (Cell Ranger, Optimus, salmon alevin, kallisto bustools, dropSeqPipe, scPipe, zUMIs, celseq2 and scruff) for scRNAseq data.

They tested these methods on datasets originated from CEL-seq2 (plate-based) and 10X Chromium (droplet-based) technologies. They focused their analytical results on computational performance, gene quantification, and gene biotype detection, along with performance assessment in downstream analyses, more specifically in normalization methods, identification of highly variable genes (HVGs), and cell clustering. They claim they have benchmarked more than 2,000 pipelines, results of combining different datasets, preprocessing workflows and downstream analysis methods. Their findings demonstrate that the selection of preprocessing workflow does not carry a big impact on scRNAseq analysis, with relatively minor differences among these combinations. These differences could even change with the most up-to-date versions of the selected software (i.e. Cell Ranger version 6.0.0 instead of v3.0.2).

*Response: We thank the reviewer for their interest in our work and constructive feedback. We agree that the use of different software versions will influence the results, and have updated our analyses to make use of newer versions of Cell Ranger (updated from v3.0.2 to v6.0.0), Optimus (updated from v1.3.6, and v2.0.0 to v4.3.2), salmon (updated from v1.0.0 to v1.5.2) and zUMIs (updated from v2.5.5 to v2.9.7). All results presented are now based on these updated software versions.*

The study motivation and importance, the standard steps of a preprocessing workflow, some differences among the selected software, the current state of research, and limitations of previous benchmarking studies are clearly and orderly stated in the introductory "background" section of the paper. Because the topic discussed in this paper revolves around UMI-based methods, UMIs themselves and their role in scRNAseq could be further described in the paragraphs where they are first mentioned (p.2, lines 14-16).

*Response: Thanks for the feedback. We have added more background on UMIs in the introduction as follows:*

*‘UMIs are random oligonucleotide barcodes of fixed length that are used to distinguish between the original molecules present in the cell and the PCR amplified copies generated during library construction. The process of UMI 'de-duplication’ is a key part of transcript quantification in scRNA-seq analysis that aims to provide molecule counts for the expressed genes in each cell and eliminate PCR-related quantification biases.’*

The overall read of the results section feels lengthy and dense (11 pages), and the discussion could be shortened and made more concise.

*Response: Thanks for the comment. Given the large number of comparisons obtained (over 3,800), we tried our best to summarize our findings and highlight the most interesting observations in the main figures in the Results section in the revised version. We have slightly reorganised the content (moving some of the Supplementary Figure panels into Main Figures and vice versa, as suggested below) and hope that this improves readability.*

It is not clear the choice of main text figures and supplementary figures, as in some sections the supplementary figures are referenced more often and seem more important than the "main" ones (For example p.13 lines 6-36). This happens again in the clustering section, where no tSNE or UMAPs plots are shown as main figures but rather kept as supplementary and referenced broadly all over the text.

*Response: Thanks for the suggestion. The figures have been reorganized and some of the t-SNE plots on pseudogenes, and comparisons of highly variable gene selection have been moved to the main figures. We chose to keep the t-SNE plots which present the clustering results as supplementary figures because we didn’t observe any distinct differences between preprocessing workflows based on these plots.*

Throughout this section, the authors document several discrepancies between their results and other previous studies (p.5 line 48, p.6 lines 6-8, p.10, lines 43-46), for which they offer vague hypothetical reasons to explain why.

*Response: Thanks for the feedback. The reasons for our speculative answers are summarised below.*

*1) For the discrepancies in the computational performance of scruff in our study compared to the results from Wang et al. (2019), we were unable to figure out the exact reason because in their manuscript no detailed information and scripts were provided about how they performed the comparisons, hence the need to hypothesize about the potential causes of the discrepancies between our results and theirs.*

*2) For the discrepancies observed between the computational performance of Cell Ranger in our study compared to the results of Gao et al. (2021), unfortunately, there was no information provided in their manuscript on whether they used a High-Performance Computing system and specified the number of cores for each run or not.*

*3) Kallisto and salmon were found to retain more accurate information about lncRNA on bulk RNA-seq datasets (Zheng et al., 2019). The ability of different aligners to detect lncRNAs varies because of different mapping strategies and our analysis was found to vary between the datasets examined. Hence in this part of the results, we present what we observe without any further explanation as there is likely to be a technical and biological component to the differences which we were unable to tease apart further.*

Chosen datasets are not representative of common scRNAseq datasets used nowadays. They do not portray big cellular heterogeneity, and more real sequenced tissues with annotated cells should have been compared.

*Response: Datasets profiling cells from mouse lung tissue were included to represent more biological heterogeneity. In our revised manuscript we have added two new datasets which profiled human PBMCs with 5K and 10K cells respectively so that results from additional datasets with more cellular heterogeneity than the simple cell line mixture experiments are presented.*

When assessing computational resources, the largest total number of reads is only 108, which falls short for most widely used datasets nowadays. This is admitted in the Discussion section (p.17, lines 20-24).

*Response: Thanks for the valuable suggestion. In our revised manuscript, we have increased the largest number of reads to 600 million for droplet-based datasets so that scalability can be better assessed.*

Suggestions:

* In the methods section (page 19), they could provide a table indicating the QC threshold applied to each of the datasets, allowing reproducibility.

*Response: We provide supplementary table 3 which presents the QC thresholds used.*

* The names of "single cell dataset" and "tissue dataset" are confusing since both are composed of single cell experiments.

*Response: We have renamed ‘single cell datasets’ to ‘cell line datasets’, and ‘tissue datasets’ to ‘lung tissue datasets’. The new PBMC datasets are called ‘PBMC datasets’. We hope that this new naming scheme is less confusing to readers.*

* The metric silhouette width is not explained, while other metrics used here for clustering assessment (ECA, ECP and ARI) are. Also, should their formulas be shown in the Methods section?

*Response: The silhouette width metric is now explained in the Methods section of the revised manuscript and the relevant formulas have also been added.*

* I've personally found hard to compare values between figures S15A and S12A, due to the design of these tile plots.

*Response: Heatmaps are now plotted that only facet the results by clustering method. Information on normalization methods is no longer included, which we hope makes the plots clearer and easier to interpret.*

* The tile plots in Fig.7 (7C and 7D) should contain rank values, so the reader can make not only a qualitative comparison depending on the color but also a quantitative one.

*Response: We have added the rank values to the updated version of this figure.*

* Table 1 is oversimplistic and vague. For example, it is not explained what constitutes a "User-friendly" software, when a YouTube tutorial for dropSeqPipe is mentioned but not the whole amount of documentation and tutorial behind Cell Ranger webpage.

*Response: Thanks for the feedback. The aim of Table 1 is to provide an overview of the advantages and limitations of the workflows we selected. We have taken on board your suggestion though and now explain different workflow aspects in more detail (e.g. why some workflows are labeled ‘user-friendly’).*

Errors and typos:

p.6 lines 33-35: The sentence is weirdly written.

p.11 line 61: They're referencing figure S8B, not S7B.

p.14 line 34: onserved instead of conserved.

p.17 line 8: repeated to particle.

p.20 lines 31-34: is weirdly written.

p.20 line 52: the middle sentence is bad written.

p.20 line 62: the last sentence is bad written.

p.21 line 17-18: Which "other figures"? This is very vague.

*Response: Thanks for the summary of errors and typos, which have been corrected in the updated version.*

Reviewer #2:

In this manuscript, the authors have benchmarked various preprocessing workflows for UMI-based single-cell RNA sequencing technologies. Overall the manuscript is excellently written and provides important insights into which methods are important for specific use cases. I have a few minor comments which can improve the clarity and impact of the paper on the broader community:

Comments:

* The motivation to use CEL-seq1/2 technology for benchmarking should be clarified, currently the motivation to benchmark with only CEL-seq for the well-based method is unclear.

*Response: We thank the reviewer for their interest in our work and constructive feedback. We chose the CEL-seq2 platform to make use of the custom in-house dataset with cell group labels that we generated using this protocol in a previous study. So it was largely out of convenience that we chose this platform as a ‘representative’ plate-based method.*

* It's unclear why authors chose to not benchmark alevin on CEL-seq data, especially when they mention in Page 3 Line 60, that alevin can quantify CEL-seq data.

*Response: Thanks for pointing this out. When we commenced our study, we were not aware that this option was available for alevin (it wasn’t documented in the tutorial (https://salmon.readthedocs.io/en/latest/alevin.html) at the time we looked into it while planning the study) - this is the only reason we didn’t include it.*

* Figure1: Salmon alevin can perform both pseudoalignment and end-to-end alignment, hence I am unclear about putting alevin into the category of pseudoaligners.

*Response: Thanks for pointing this out. We have clarified the strategy we selected for running salmon alevin in the results section. Alevin is applied in the mode of selective alignment to full genome decoys. We also include results from alevin-fry which is run in pseudoalignment to splici (spliced and intron) reference mode.*

* Figure2: As more recent single-cell technologies can profile millions of cells it's worth checking how computational tools scales when given more resources. Henceforth it'd be worth comparing the scalability of the tools wrt number of threads.

*Response: Thanks for the suggestion - we now compare the scalability of selected workflows on droplet-based datasets and include these results in Figure 2C.*

* Figure 3F It's unclear why is there no kallisto based map ? I know authors chose to exclude kallisto from benchmark but having the umap based on kallisto quantified data makes the case even stronger if it's not separating the cells based on cell-lines.

*Response: Good point. We now include a GLMPCA plot for kallisto bustools in Figure S2C.*

* On Page 8 Line 9: Authors mention "Calculating correlations with intron counts excluded by zUMIs didn't noticeably increase the correlation values" but from Figure S3F, it seems the overall correlation seems to increase, no ?

*Response: Thanks for pointing out this inconsistency between the text and the results. We have re-written this sentence as follows:*

*‘Calculating correlations with intron counts excluded noticeably increases the correlation values (Additional file 2: Figure S4D), which suggests that the addition of intron counts is potentially adding noise to the results.’ (note the updated figure order in the revised version)*

* Figure 4A (10xv2_tissue2) The results from alevin pipeline is unclear as the number of cells are significantly high. It's worth checking the command line flags used for quantifying the data, specially `--expectCells` flag which can be provided to improve the number of cells, as also provided to the Cellranger pipeline.

*Response: The reason why we did not apply -expectCells is that we would like to apply emptyDrops which expects the full, unfiltered count matrices, so --keepCBFraction 1 was applied instead. In the updated version, to avoid considerably more cells being retained by emptyDrops, the filtering strategy from 10xv2 was applied, which returns a reasonable number of cells.*

* Figure 5(B-C, E-H): Since most of the lines in the figures overlap, it's hard to interpret it. Also the point of having solid lines versus dotted lines is unclear. I think the figure can be interpreted better with disjoint plots of each tool, like Violin Plots.

*Response: Thanks for the suggestion. We have modified the density plots so that they all have solid curves and also provided a version of the plot that is faceted by workflow so that the densities can be seen more clearly without overplotting (Figure S5B).*

* Color coding of Fig S4D is not clear, for example, what is the annotation of the pink color ?

*Response: Thanks for pointing this out. In the updated version of this Figure (now S6F), colour legends have been added.*

* Page 10 line 52, Authors mention "These results suggest that focusing analysis efforts on the signal from protein coding genes in datasets that profile complex tissues with greater cell type diversity may be an optimal strategy." Are they talking about pseudoalignment methods specifically or in general for all other methods?

*Response: Apologies for the confusion - we meant in general for all methods here, and have added ‘for all preprocessing methods’ to the end of this sentence to make it clearer.*

* Page 11 line 61: Authors mention PCA plots on Fig7B, but it seems they are Violin plots, I am guessing this one is a typo.

*Response: Thanks for pointing out this mistake, which we have fixed.*

Optional Comments: Based on authors discretion they can choose or not to perform the analyses, but it can improve the overall impact of the paper:

* Multimodal data benchmarking: Advent of multimodal technologies have enabled measurement of multiple single-cell modalities within a single-cell. For example CITE-seq can profile both cell-surface proteins and RNA-seq on a droplet based single-cell system. The biggest advantage of these methods is the availability of robust protein measurements methods, which can be used to cluster cells and the second modality such as RNA-seq can be validated against. It'd be interesting to know how different scRNA-seq preprocessing workflows trades off compared to a clustering based on protein data.

*Response: This is a very interesting suggestion and would be a great aim for a future study. We agree on the advantage you mention about CITE-seq datasets that cover both protein and RNA information and have found that being able to measure isoform-specific expression at the single-cell level can help improve the correlation between these quantities (see Tian et al. bioRxiv https://www.biorxiv.org/content/10.1101/2020.08.10.243543v1, Figure 3G).*

* New generation of methods: Recently the authors of SCtransform and alevin have released newer version of their software: SCtransform 2 (Satija Lab) and alevin-fry (Patro Lab). Although both the methods are not yet peer-reviewed (only preprinted), it'd still be interesting to know how these methods stand in the current benchmarks.

*Response: Thanks for these suggestions. In our updated manuscript, we have included results for sctransform 2 (named sctransform glmGamPoi in our study - we hope this is the version you were referring to) and alevin-fry and also updated the version numbers of other methods applied to more recent ones and re-run all analyses based on these newer versions.*

**Second round of review**

**Reviewer 1**

The authors have done significant work to improve the manuscript. They answered all questions and addressed problematic points I commented on last time, including a dataset with higher heterogeneity (10x's PBMC) and using the latest software versions. I consider that the manuscript is now of a very high quality and relevant for the single cell community.

**Reviewer 2**

All my comments have been addressed. Congratulations to the authors for the fantastic work.
